# Mendelian randomization reveals association of gut microbiota with Henoch–Schönlein purpura and immune thrombocytopenia

**DOI:** 10.1007/s12185-024-03777-1

**Published:** 2024-04-26

**Authors:** Chendong Jiang, Shu Deng, Xiaohan Ma, Juan Song, Jinpeng Li, Enwu Yuan

**Affiliations:** 1 Department of Laboratory Medicine, The Third Affiliated Hospital of Zhengzhou University, Zhengzhou, China; 2 Department of Medical Imaging, The Third Affiliated Hospital of Zhengzhou University, Zhengzhou, China; 3 Zhengzhou Key Laboratory for In Vitro Diagnosis of Hypertensive Disorders of Pregnancy, Zhengzhou, China

**Keywords:** Mendelian randomization, Gut microbiota, Causal relationship, Immune thrombocytopenia, Secondary thrombocytopenia, Henoch–Schönlein purpura

## Abstract

Gut microbiota have been linked to immune thrombocytopenia (ITP) and Henoch–Schönlein purpura (HSP) in recent studies, but a cause-and-effect relationship is unclear. We used Mendelian randomization (MR) to assess causal relationships between gut microbiota and HSP/ITP using summary statistics from the GWAS dataset of the international MiBioGen and FinnGen consortium. The IVW method was used as the main evaluation indicator. MR analysis of 196 intestinal flora and HSP/ITP/sTP phenotypes showed that 12 flora were potentially causally associated with ITP, 6 with HSP, and 9 with sTP. The genes predicted that genus Coprococcus3 (*p* = 0.0264, OR = 2.05, 95% CI 1.09–3.88)and genus Gordonibacter (*p* = 0.0073, OR = 1.38; 95% CI 1.09–1.75) were linked to a higher likelihood of developing ITP. Additionally, family Actinomycetaceae (*p* = 0.02, OR = 0.51, 95% CI 0.28–0.90) and order Actinomycetales (*p* = 0.0199, OR = 0.50, 95% CI 0.28–0.90) linked to reduced HSP risk. Genus Ruminococcaceae UCG013 (*p* = 0.0426, OR = 0.44, 95% CI 0.20–0.97) negatively correlated with sTP risk. Our MR analyses offer evidence of a possible cause-and-effect connection between certain gut microbiota species and the likelihood of HSP/ITP.

**Supplementary Information:**

The online version contains supplementary material available at 10.1007/s12185-024-03777-1.

## Introduction

HSP and ITP are both prevalent immune-mediated bleeding disorders characterized by distinct symptoms of purpura [1, 2]. ITP, also known as idiopathic thrombocytopenic purpura or immune thrombocytopenic purpura, is a condition characterized by a low platelet count caused by autoantibodies targeting platelet antigens [3]. ITP is a hematologic disorder that is relatively common, with the highest incidence observed in the pediatric population [4]. Approximately 40% of cases occur in children under 10 years of age [5]. The estimated overall incidence of ITP in the United States is approximately 3.3 cases per 100,000 persons per year [3]. The incidence of ITP in pregnant women ranges from 1 to 10 per 10,000 cases. ITP accounts for approximately 1–4% of all cases of thrombocytopenia during pregnancy and is the primary cause of thrombocytopenia in the early stages of pregnancy [6]. The primary clinical symptom of ITP is hemorrhage in the skin and mucous membranes [7]. The primary clinical symptom of ITP is hemorrhage in the skin and mucous membranes [8]. It is generally believed that the abnormal immune response, which mediates platelet destruction, and the disorder of platelet production caused by the impairment of bone marrow megakaryocyte maturation and poor platelet production, play a key role in the pathogenesis [9]. Early treatment does not reduce the incidence of chronic ITP, but it can help restore platelet levels quickly, improve quality of life, and reduce the occurrence of severe bleeding or complications [10]. In Henoch–Schönlein purpura (HSP) or immunoglobulin A vasculitis (IgA), small blood vessels in the skin, kidneys, and gastrointestinal system are affected [1].This condition is classified as a small-vessel leukocytoclastic vasculitis resulting from the deposition of immune complexes. It can present as either a systemic or localized disease, with commonly affected organs including the skin, kidneys, gastrointestinal system, and joints [1]. With a prevalence of 20.4 per 100,000, HSP is the most common form of systemic vasculitis in children [11].

There is mounting evidence that the gut microbiota plays a crucial role in maintaining metabolic and immune system balance [12–14]. Dysregulation of the gut microbiota can contribute to the development of autoimmune and metabolic diseases, as well as hematologic disorders such as rheumatoid arthritis (RA) [15]. In recent years, numerous studies have explored the connection between gut microbiota and various diseases, including rheumatoid arthritis (RA) [15], ankylosing spondylitis (AS) [16], inflammatory bowel disease (IBD) [17], immune disorders like systemic lupus erythematosus (SLE) [18], and metabolic disorders such as type II diabetes mellitus [19] and atherosclerotic cardiovascular disease (ACVD) [20]. Changes in the abundance of gut microbiota, such as Methylobacterium, Sphingomonas, and Staphylococcus, can impact serum salicylate levels. The latter has been found to inhibit platelet production in megakaryocytes and induce the development of ITP. The activation status of platelets in patients with ITP has been associated with gut flora [21].

Xiaomin Yu et al. discovered that specific lipid metabolites, including RvD2, eicosatetraenoic acid, monooleic acid, and phosphatidylcholine, were up-regulated in the gut microbiota of patients with ITP. They also found that these metabolites could regulate platelet homeostasis, reduce platelet activation, and consequently impact platelet function [22]. Furthermore, the microbiota can affect the effectiveness of ITP treatment. Research has shown that the microbiota found in the airways of asthma patients can cause resistance to corticosteroids [23]. The study revealed significant differences in the gut microbiota of patients with corticosteroid-resistant ITP compared to both healthy individuals (3 genera and 13 species) and patients with corticosteroid-sensitive ITP (21 genera and 59 species). This indicates that the imbalance of microbiota differs between corticosteroid-sensitive and drug-resistant ITP patients [24]. Disturbed intestinal flora is found in children with HSP. This could play a role in the onset of HSP by impacting the production and breakdown of unsaturated fatty acids [25–27].

While there is a connection between gut flora and ITP, there is a lack of comprehensive data regarding the link between gut microbiota and ITP. It is crucial to rule out any secondary causes of thrombocytopenia for an accurate clinical diagnosis of ITP. Therefore, exploring the association between gut microbiota and secondary thrombocytopenia (sTP) could provide valuable understanding. To identify specific pathogenic bacterial taxa and screen for genetic variants, we utilized genome-wide association study (GWAS) data and employed MR analysis [28]. Furthermore, we examined the possible cause-and-effect connection between the gut microbiota and disease susceptibility genes like HSP, ITP, and sTP.

## Study design and methods

### Study design

**Fig. 1:**
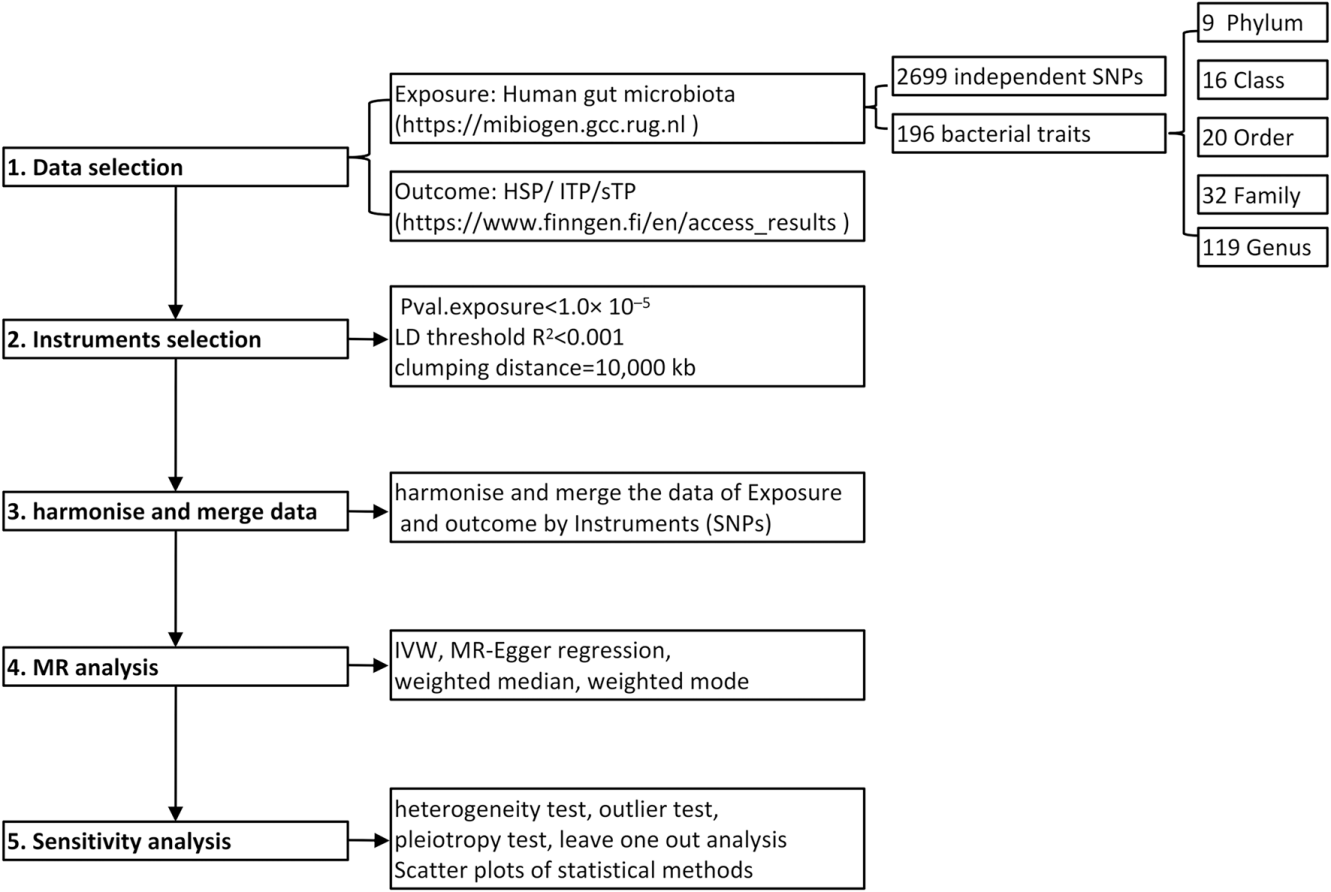
Abbreviated diagram of the Mendelian randomization study design. Abbreviations: MR, Mendelian randomization; IVW, Inverse variance weighted; SNP, single nucleotide polymorphism

A summary of the study's design is depicted in Fig. 1. This study strictly adheres to the three hypotheses of MR analyses: the association hypothesis, which posits that instrumental variables (IVs) are strongly correlated with exposure. The independent hypothesis states that IVs are not influenced by confounding variables between the exposure and the outcome. IVs only have an effect on the outcome through exposure and do not directly affect the exclusive hypothesis of the outcome.

### Data sources of gut microbiota

The information on the microbiome of the human gut was collected from the GWAS dataset of the MiBioGen International Consortium [28]. The research combined extensive, multiethnic 16S ribosomal RNA gene sequencing and genotyping data from 24 different groups, including 18,340 participants in the United States, United Kingdom, Germany, Finland, Denmark, the Netherlands, Canada, and South Korea. The data were adjusted for major components such as age, sex, technical covariates, and study population. Upon analysis of the data, 15 taxa of bacteria with unknown or unspecified names were excluded, resulting in the inclusion of 196 taxa, comprising 119 genera, 32 families, 20 orders, 16 classes, and 9 phyla. The data for this MR analysis were derived from publicly available GWAS genetic aggregate data, thus obviating the need for additional ethical approval or consent for participation.

### Data sources for HSP/ITP/sTP

The aggregated outcomes data are sourced from data R9 published by the FinnGen Consortium. HSP included 856 cases and 391,567 controls, ITP included 810 cases and 391,613 controls, and sTP included 298 cases and 392,125 controls. Detailed information about the outcome data from the included studies is summarized in Table 1. In 810 cases of ITP patients, females accounted for 52.7% (427 cases) and males accounted for 47.3% (383 cases).The mean age at first event for females was 43.7 years, and for males it was 54.73 years. The death rate for the entire study period, spanning from 1998 to 2019, was 0.08, with a Hazard Ratio (HR) and a 95% Confidence Interval (CI) of 5.61 (3.19, 9.86). Furthermore, the death rates at both the 15-year and 5-year marks were both 0.01, with HR (95% CI) values of 2.08 (1.20, 3.62) and 5.43 (3.55, 8.30) respectively. In 856 cases of HSP patients, females accounted for 59.1% (506 cases) and males accounted for 40.9% (350 cases). The mean age at first event for females was 36.25 years, and for males it was 43.10 years. The death rate for the entire study period from 1998 to 2019 was calculated to be 0.13,with HR (95% CI) of 9.04 (5.81, 14.06). Additionally,the death rates at both the 15-year and 5-year marks were determined to be 0.02 and 0.01 respectively,with HR (95% CI) values of 3.23 (1.95, 5.36) and 6.71 (3.91, 11.51). In 298 cases of sTP patients, it was found that females accounted for 52.7% (157 cases) and males accounted for 47.3% (141 cases). The mean age at the first event for females was approximately 53.73 years, while for males it was approximately 59.08 years old.

**Table 1:** Data sources and traits for MR analysis

Characteristic	Data source	Sample size	Cases	Population	Year
Gut microbiota	MiBioGen Consortium	602,604	129,080	European	2021
HSP	FinnGen study	372,360	856	European	2022
ITP	FinnGen study	372,314	810	European	2022
sTP	FinnGen study	371,802	298	European	2022

*HSP* Henoch–Schönlein purpura; *ITP* Immune thrombocytopenia; *sTP* secondary thrombocytopenia

### Instruments selection

The selection criteria and steps for IVs were as follows: to obtain a satisfactory number of IVs, we selected *p* < 1.0 × 10^–5^ as the threshold to screen for significantly correlated single nucleotide polymorphisms (SNPs). 2. We used the "TwoSampleMR" package to remove linkage disequilibrium by setting the linkage disequilibrium (LD) threshold to R2 < 0.001 and the aggregation distance to 10,000 kb. 3. The distance is 10,000 kb. 3. When quantifying using the *F*-statistic, we set *F* > 10 to exclude weak instrumental variables. To calculate the *F*-value, we extracted the beta, standard error (SE), and *p* value (*p*) for each SNP. The *F*-value was calculated as *F* = Beta^2^/SE^2^.

### Statistical analysis

In this study, four methods, namely IVW, MR-Egger regression, weighted median, and weighted mode, were utilized to investigate the potential causal relationship between the gut microbiota and HSP, ITP, and sTP. The IVW method served as the primary analytical approach. The weighted median approach operated under the assumption that more than 50% of the IVs were plausible. To address potential horizontal pleiotropy, concerns were evaluated through intercept tests for MR-Egger regression, Mendelian Randomization Pleiotropy Residual Sum and Outlier (MR-PRESSO) analysis, and sensitivity analyses. The reliability of MR results may be compromised if the *p*-value of the intercept test in MR-Egger regression is less than 0.05. If the *p*-value of MR-PRESSO is less than 0.05, it is necessary to identify outliers and remove them from the IVs before conducting MR analysis. In addition, we tested for heterogeneity among the included data SNPs using Cochran's *Q* value. If a *p*-value is less than 0.05, it indicates the presence of heterogeneity. In this case, we opt for the random-effects IVW approach. Conversely, if the *p*-value is greater than or equal to 0.05, we use a fixed-effects model analysis. Finally, the relationships between human gut microbiota and diseases were quantified as odds ratios (OR) and their corresponding 95% confidence intervals (CI).

To assess causality more rigorously, we adjusted for the number of bacterial taxa associated with each attribute using the Bonferroni method, which required setting more stringent significance *p*-values. For example, we used the following adjusted *p*-values: Door: 0.05/9 (5.6 × 10^–3^), Class: 0.05/16 (3.1 × 10^–3^), Family: 0.05/32 (1.56 × 10^–3^), and Genus: 0.05/119 (4.20 × 10^–4^). If the *p*-value of the results of this MR analysis falls between the corrected threshold and 0.05, we would consider the bacterial taxon as providing suggestive evidence of a potential causal association with the disease and performed a reverse MR analysis. The MR analyses were conducted using R 3.6.3 software and the "TwoSampleMR" and "MR-PRESSO" packages. The code for the study is provided in Supplementary Table 1.

## Results

In this MR analysis of gut flora with HSP/ITP/sTP, F values were calculated for 196 gut microbial SNPs, with the F-statistics for SNPs ranging from 12.6 to 32.5, all of which were above 10, indicating a lower likelihood of experiencing weak instrument bias (Supplementary Table 2).

### Causal effects of gut microbiota on ITP

**Fig. 2:**
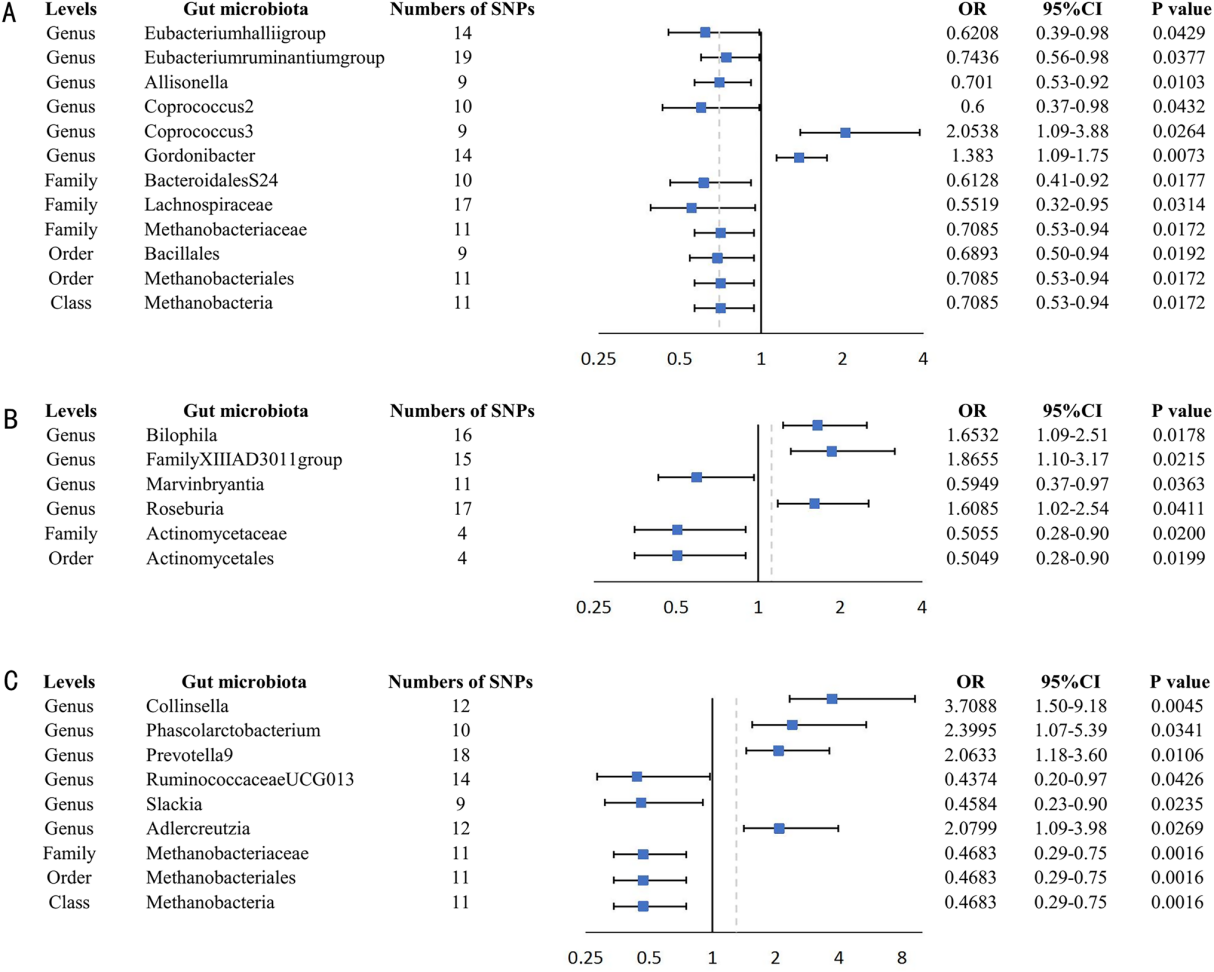
Causal estimation of gut microbiota and disease. **A** MR results of Gut microbiota versus ITP. **B** MR results of Gut microbiota versus HSP. **C** MR results of Gut microbiota versus sTP. Abbreviations: SNP, single nucleotide polymorphism; CI, confidence interval; OR, odds ratio

This research uncovered 12 causal connections between the gut microbiota and ITP (Supplementary Table 3). The genetically forecasted genus Coprococcus3 (*p* = 0.0264, OR = 2.05, 95% CI 1.09–3.88) and genus Gordonibacter (*p* = 0.0073, OR = 1.38, 95% CI 1.09–1.75) exhibited positive associations with the likelihood of ITP as determined by the IVW method (Fig. 2). The connection between genus Coprococcus3 (*p* = 0.0360, OR = 2.45, 95% CI 1.06–5.66) and genus Gordonibacter (*p* = 0.025, OR = 1.41, 95% CI 1.04–1.89) and ITP remained steady in the weighted-median method. Furthermore, we observed that five bacterial characteristics were tentatively linked to a reduced risk of ITP in the IVW method (*p* = 0.0172, OR = 0.71, 95% CI 0.53–0.94 for class Methanobacteria; *p* = 0.0177, OR = 0.61, 95% CI 0.41–0.92 for family BacteroidalesS24.7 group; *p* = 0.0314, OR = 0.55, 95% CI 0.32–0.95 for family Lachnospiraceae; *p* = 0.0172, OR = 0.71, 95% CI 0.53–0.94 for family Methanobacteriaceae; *p* = 0.0429, OR = 0.62, 95% CI 0.39–0.98 for genus Eubacteriumhalliigroup; *p* = 0.0377, OR = 0.74, 95% CI 0.56–0.98 for genus Eubacteriumruminantiumgroup; *p* = 0.0103, OR = 0.70, 95% CI 0.53–0.92 for genus Allisonella; *p* = 0.0432, OR = 0.60, 95% CI 0.37–0.98 for genus Coprococcus2; *p* = 0.0192, OR = 0.69, 95% CI 0.50–0.94 for order Bacillales; *p* = 0.0172, OR = 0.71, 95% CI 0.53–0.94 for order Methanobacteriales). However, the results from the weighted median method did not support such a causal effect. The MR-Egger and MR-PRESSO tests indicated no evidence of horizontal pleiotropy or outliers (*p* > 0.05). Furthermore, the results of Cochrane's *Q*-test revealed no significant heterogeneity (*p* > 0.05) (Table 2). Additionally, leave-one-out analyses demonstrated that none of the identified causal associations were affected by any individual independent variable (Supplementary Fig. 1). None of the outcomes from Bonferroni's corrected test indicated the presence of a causal effect.

**Table 2:** Comparative analysis using MR to investigate the relationship between gut microbiota and HSP/ITP/sTP, incorporating assessments for horizontal pleiotropy and heterogeneity

Outcome	Exposure	SNP	IVW (95%CI)	P_IVW	Egger_intercept	SE	P_MR-Egger	Cochran’s Q	P_Cochran’s Q
ITP	Genus Eubacteriumhalliigroup	14	0.6208 (0.3913, 0.9849)	0.0429	− 0.0120	0.0413	0.7769	13.9319	0.3787
	Genus Eubacteriumruminantiumgroup	19	0.7436 (0.5624, 0.9833)	0.0377	− 0.0004	0.0461	0.9940	9.3626	0.9507
	Genus Allisonella	9	0.701 (0.5344, 0.9196)	0.0103	− 0.1798	0.1311	0.2128	7.8403	0.4492
	Genus Coprococcus2	10	0.6 (0.3658, 0.9846)	0.0432	0.0626	0.1033	0.5610	2.8257	0.9708
	Genus Coprococcus3	9	2.0538 (1.0878, 3.8775)	0.0264	0.0317	0.1112	0.7840	4.4147	0.8179
	Genus Gordonibacter	14	1.383 (1.091, 1.7537)	0.0073	0.0124	0.0710	0.8648	4.2947	0.9876
	Family BacteroidalesS24.7group	10	0.6128 (0.4089, 0.9185)	0.0177	− 0.0932	0.0843	0.3012	7.6144	0.5734
	Family Lachnospiraceae	17	0.5519 (0.3211, 0.9484)	0.0314	0.0789	0.0564	0.1821	19.0125	0.2680
	Family Methanobacteriaceae	11	0.7085 (0.5336, 0.9407)	0.0172	0.0460	0.0843	0.5990	6.0130	0.8142
	Order Bacillales	9	0.6893 (0.5048, 0.9411)	0.0192	− 0.1060	0.1017	0.3321	10.2473	0.2481
	Order Methanobacteriales	11	0.7085 (0.5336, 0.9407)	0.0172	0.0460	0.0843	0.5990	6.0130	0.8142
	Class Methanobacteria	11	0.7085 (0.5336, 0.9407)	0.0172	0.0460	0.0843	0.5990	6.0130	0.8142
HSP	Genus Bilophila	16	1.6532 (1.0907, 2.5057)	0.0178	0.0597	0.0643	0.3688	7.6392	0.9374
	Genus FamilyXIIIAD3011group	15	1.8655 (1.09619, 3.1748)	0.0215	− 0.0840	0.1063	0.4432	19.9880	0.1305
	Genus Marvinbryantia	11	0.5949 (0.3658, 0.9674)	0.0363	− 0.0736	0.0771	0.3649	7.7369	0.6545
	Genus Roseburia	17	1.6085 (1.01936, 2.5383)	0.0411	0.0119	0.0475	0.8049	12.1242	0.7354
	Family Actinomycetaceae	4	0.5055 (0.2844, 0.8983)	0.0200	0.0279	0.0800	0.7607	2.3360	0.5057
	Order Actinomycetales	4	0.5049 (0.284, 0.8976)	0.0199	0.0285	0.0799	0.7553	2.3250	0.5070
sTP	Genus Collinsella	12	3.7088 (1.4981, 9.1816)	0.0045	0.0615	0.1269	0.6382	10.0675	0.5243
	Genus Phascolarctobacterium	10	2.3995 (1.0678, 5.392)	0.0341	− 0.0504	0.1389	0.7264	3.5592	0.9380
	Genus Prevotella9	18	2.0633 (1.1839, 3.5958)	0.0106	− 0.0666	0.0757	0.3923	19.0501	0.3257
	Genus RuminococcaceaeUCG013	14	0.4374 (0.1967, 0.9728)	0.0426	0.0217	0.0825	0.7967	6.5780	0.9226
	Genus Slackia	9	0.4584 (0.2334, 0.9004)	0.0235	− 0.1095	0.1718	0.5443	5.2656	0.7288
	Genus Adlercreutzia	12	2.0799 (1.0874, 3.9781)	0.0269	0.1310	0.1496	0.4017	9.4715	0.5785
	Family Methanobacteriaceae	11	0.4683 (0.2926, 0.7494)	0.0016	− 0.1419	0.1397	0.3361	5.0523	0.8877
	Order Methanobacteriales	11	0.4683 (0.2926, 0.7494)	0.0016	− 0.1419	0.1397	0.3361	5.0523	0.8877
	Class Methanobacteria	11	0.4683 (0.2926, 0.7494)	0.0016	− 0.1419	0.1397	0.3361	5.0523	0.8877

*HSP* Henoch–Schönlein purpura; *ITP* Immune thrombocytopenia; *sTP* secondary thrombocytopenia; *SNP* single nucleotide polymorphism; *SE* standard error; *MR* Mendelian randomization; *IVW* Inverse Variance Weighted

### Causal effects of gut microbiota on HSP

This research uncovered six causal connections between the gut microbiota and HSP (Supplementary Table 4). The genetically forecasted family Actinomycetaceae (*p* = 0.0200, OR = 0.51, 95% CI 0.28–0.90) and order Actinomycetales (*p* = 0.0199, OR = 0.50, 95% CI 0.28–0.90;) exhibited negative associations with the likelihood of HSP as determined by the IVW method (Fig. 2). The connection between family Actinomycetaceae (*p* = 0.0236, OR = 0.45, 95% CI 0.23–0.90), order Actinomycetales (*p* = 0.0341, OR = 0.45, 95% CI 0.21–0.94), and HSP remained steady in the weighted-median method. In addition, the increased presence of the genetically predictive genus Bilophila (*p* = 0.0178, OR = 1.65, 95% CI 1.09–2.51), genus FamilyXIIIAD3011group (*p* = 0.0215, OR = 1.87, 95% CI 1.10–3.17) and genus Roseburia (*p* = 0.0411, OR = 1.61, 95% CI 1.02–2.54) were found to be linked to the development of HSP using the IVW method. Conversely, the presence of genus Marvinbryantia (*p* = 0.0363, OR = 0.59, 95% CI 0.37–0.97) was associated with a decreased likelihood of HSP occurrence. Nevertheless, the findings obtained from the weighted median method did not provide evidence for a causal effect. The MR-Egger and MR-PRESSO tests indicated no evidence of horizontal pleiotropy or outliers (*p* > 0.05). Furthermore, the results of Cochrane's Q-test revealed no significant heterogeneity (*p* > 0.05) (Table 2). Additionally, leave-one-out analyses demonstrated that none of the identified causal associations were affected by any individual independent variable (Supplementary Fig. 2). None of the outcomes from Bonferroni's corrected test indicated the presence of a causal effect.

### Causal effects of gut microbiota on sTP

This research uncovered 9 causal connections between the gut microbiota and sTP (Supplementary Table 5). The genetically forecasted genus RuminococcaceaeUCG013 (*p* = 0.0426, OR = 0.44, 95% CI 0.20–0.97), family Methanobacteriaceae (*p* = 0.0016, OR = 0.47, 95% CI 0.29–0.75), order Methanobacteriales (*p* = 0.0016, OR = 0.47, 95% CI 0.29–0.75), class Methanobacteria (*p* = 0.0016, OR = 0.47, 95% CI 0.29–0.75) exhibited negative associations with the likelihood of sTP as determined by the IVW method (Fig. 2). The association between genus RuminococcaceaeUCG013 (*p* = 0.0337, OR = 0.32, 95% CI 0.11–0.92), family Methanobacteriaceae (*p* = 0.0325, OR = 0.50, 95% CI 0.27–0.94), order Methanobacteriales (*p* = 0.0359, OR = 0.50, 95% CI 0.27–0.65), class Methanobacteria (*p* = 0.0328, OR = 0.50, 95% CI 0.27–0.95), and sTP remained steady in the weighted-median method.

Furthermore, we noted that four bacterial characteristics were tentatively linked to an increased likelihood of sTP using the IVW method ( *p* = 0.0045, OR = 3.71, 95% CI 1.50–9.18 for genus Collinsella; *p* = 0.0341, OR = 2.40, 95% CI 1.07–5.39 for genus Phascolarctobacterium; *p* = 0.0107, OR = 2.06, 95% CI 1.18–3.60 for genus Prevotella9; *p* = 0.0269, OR = 2.08, 95% CI 1.09–3.98 for genus Adlercreutzia) and one bacterial trait was suggestively associated with a lower risk of sTP (*p* = 0.0235, OR = 0.46, 95% CI 0.23–0.90 for genus Slackia). Nevertheless, the findings obtained from the weighted median method did not provide evidence for a causal effect. The MR-Egger and MR-PRESSO tests indicated no evidence of horizontal pleiotropy or outliers (*p* > 0.05). Furthermore, the results of Cochrane's Q-test revealed no significant heterogeneity (*p* > 0.05) (Table 2). Additionally, leave-one-out analyses demonstrated that none of the identified causal associations were affected by any individual independent variable (Supplementary Fig. 2). None of the outcomes from Bonferroni's corrected test indicated the presence of a causal effect.

Finally, our inverse MR analysis using the IVW method did not reveal any potential inverse association of gut flora with these three diseases.

## Discussion

Our research employed magnetic resonance analysis and comprehensive genome-wide association study (GWAS) data to explore potential causal connections between gut microbiota and HSP, ITP, and sTP at the genetic prediction level, yielding findings with strong causal explanatory capability. This two-sample MR investigation identified a combined total of 12 bacterial taxa potentially linked to the susceptibility of ITP, 6 bacterial taxa associated with the susceptibility of HSP, and 9 bacterial taxa associated with the susceptibility of sTP. Given the potential for bias in IVW analyses, additional MR methods may help to identify causal relationships. Our sensitivity analyses, using various MR methods and restricted IV groups, found that two bacterial taxa, genus Coprococcus3 and genus Gordonibacter, were associated with the risk of ITP. Additionally, two bacterial taxa, such as order Methanobacteriales and class Methanobacteria, were associated with the risk of HSP. Four bacterial taxa, including genus RuminococcaceaeUCG013, family Methanobacteriaceae, order Methanobacteriales, and class Methanobacteria, were found to be correlated with sTP. In addition, MR analyses between gut flora and sTP and ITP identified the presence of the same instrumental variables and bacterial taxa with potential causal associations, such as family Methanobacteriaceae order, order Methanobacteriales, and class Methanobacteria, have all been shown to have a negative correlation with sTP and ITP. This indicates that there is a common mechanism or potential interference in the correlation between Methanobacteria and ITP and sTP.

Our study revealed that genus Coprococcus3 (OR: 2.05; 95% CI 1.09, 3.88; *p* = 0.0264) and genus Gordonibacter (OR: 1.38; 95% CI 1.09, 1.75; *p* = 0.0073) were positively associated with the risk of ITP. Coprococcus is a significant member of the family Lachnospiraceae (phylum Firmicutes), an important genus of intestinal bacteria, with most of the strains being isolated from feces. It is also a significant producer of butyric acid. In a research conducted on a cohort of middle-aged individuals from Southwest China, it was observed that there was an inverse relationship between the abundance of Firmicutes and the levels of IgM. Additionally, the ratio of Firmicutes/Bacteroidetes was found to have a positive correlation with IgG and IgM levels [29]. It has been reported that the abundance of the phylum. Firmicutes and the ratio of Firmicutes/Bacteroidetes are correlated with Th17-Tregs anti-differentiation, which is regulated by IL-6 [30]. These findings suggest that they play a crucial role in regulating the immune homeostasis of systemic inflammation [31]. Upregulation of IL-6 has been associated with autoimmune diseases such as rheumatoid arthritis, multiple sclerosis, and asthma [22, 32].Additionally, a negative correlation was found between Th17 cells and elevated levels of Roseburia and Coprococcus in patients with primary Sjögren's syndrome [33]. An elevated presence of Coprococcus has been noted in female HLA-B27 positive patients compared to healthy controls [34]. Additionally, Coprococcus was found to be lower in the IgA nephropathy group but higher in the IgA vasculitis group [35]. The mechanisms through which Coprococcus3 may impact ITP could involve several factors. It has been demonstrated that C. faecalis regulates the production of the cytokines IL-1β and IL-6, thereby coordinating the inflammatory response during infection [36]. Stimulation with Candida albicans mycelia resulted in increased levels of IL-1β and IL-6 in circulating peripheral blood mononuclear cells, and this reaction was specifically linked to Coprococcus. Additionally, this finding showed a negative correlation with the production of IL-22 induced by S. aureus [37]. In contrast, IL-6 levels were significantly higher in patients with ITP [22]. Curiously, bacteria of the genus Coprococcus have also been researched for their anti-inflammatory effects, as they help suppress immune responses and lessen the severity of allergic reactions. The unique role of Coprococcus3 in ITP warrants further investigation.

Coprococcus is strongly associated with lipid metabolism. A study of 534 healthy adult Dutch volunteers found that more than 20% of metabolites were strongly associated with platelet activation markers, with the majority being lipid-related. This suggests that the gut microbiota plays a critical role in regulating platelet function [38]. In patients with ITP, the gut microbiota regulates elevated lipid metabolites such as RvD2, eicosatetraenoic acid, monooleic acid, and phosphatidylcholine. RvD2 has been shown to reduce platelet activation and maintain platelet function. Eicosatetraenoic acid is believed to regulate platelet homeostasis. Monooleic acid and phosphatidylcholine are both considered key compounds that promote platelet activation [22]. Metabolic profiling revealed a negative correlation between steatosis and aromatic and branched-chain amino acids, as well as glycoprotein acetyl groups, along with alpha diversity and fecal cocci 3 [39]. Anaerobic Coprococcus3 was associated with a lower incidence of steatosis. In contrast, Hoyles et al. found through liver biopsy that the genus Coprococcus was associated with a lower presence of hepatocyte balloons [40]. We hypothesize that Coprococcus3 may be involved in regulating platelet activation and homeostasis by influencing lipid metabolism, but further comprehensive studies are required to confirm this.

Gordonibacter, a group of Gram-positive bacteria belonging to the family Eggerthellaceae, is capable of metabolizing dietary ellagitannins, which are hydrolyzed to ellagic acid and then into urolithin [41]. Urolithin is a dibenzopyrone metabolite that has high bioavailability and shows anti-inflammatory activity in vivo [42]. It also promotes the browning of adipocytes, enhances cholesterol metabolism, inhibits the growth of transplanted tumors, mitigates inflammation, and down-regulates neuronal amyloid formation through the β3-AR/PKA/p38MAPK, ERK/AMPKα/SREBP1, PI3K/AKT pathways [43]. However, the mechanism of Gordonibacter in ITP has not been studied. Furthermore, Lachnospiraceae, Methanobacteria, and Bacteroidales S24, which are associated with ITP, exhibit a negative correlation with ITP. This suggests that these species may offer protection against the disease. Xiaomin Yu and his team found that the Lachnospiraceae NC2004 was depleted in ITP patients, consistent with the results of this analysis. However, the genus. Bacteroides in ITP patients showed enrichment [22].

Our study revealed a potential negative correlation between family Actinomycetaceae, order Actinomycetales and the risk of developing HSP. HSP is a systemic vasculitis primarily induced by IgA-mediated immune complex deposition in the vessel wall, often involving IgG class as well. In recent research, the activation of immune cells, particularly granulocytes, results in the release of inflammatory factors [44]. At present, there was currently limited evidence to suggest a direct correlation between Actinomycetaceae and HSP. However, Actinomycetaceae may be associated with certain infectious diseases that could potentially impact platelet function. Further research was needed to explore any potential relationship between Actinomycetaceae and HSP. However, Our study revealed a potential positive correlation between family Defluviitaleaceae, genus DefluviitaleaceaeUCG011 and the risk of developing HSP. In contrast, the genus Sutterella showed a negative correlation with HSP, as determined by MR analysis using Finnish data available on the OPEN GWAS website (GWAS ID: Finn-b-d3_allergpura). The family Defluviitaleaceae and its genus Defluviitaleaceae UCG011 may have a modulating effect on granulocytes through CD11c. Neutrophil infiltration plays an important role as a mediator during vascular injury in HSP [45]. CD11c is an integrin typically expressed on dendritic cells and is involved in multiple immune responses [46, 47]. The Defluviitaleaceae UCG011 has been found to be positively associated with urticaria and granulomatous polyangiitis [46, 48]. Sutterella is the type genus of the family Sutterellaceae within the order Burkholderiales of the class β-Proteobacteria. The genus Sutterella does not directly induce significant inflammation, but rather acts by degrading IgA, affecting local or widespread immune barriers. This mechanism is implicated in the pathogenesis of ulcerative colitis [49]. A study published in Nature revealed that elevated levels of Sutterella in the gut microbiota resulted in reduced IgA levels in the feces or intestinal mucosa. The presence of Sutterella resulted in the degradation of IgA, and both live bacteria and bacterial lysates degraded the free or bound secreted components of IgA due to the production and secretion of IgA proteases [50]. We hypothesize that this may be one of the primary reasons for the negative correlation between Sutterella and HSP.

This study has some limitations. Firstly, the data analyzed was sourced from open databases and aggregated. The study population is not homogeneous, consisting mostly of individuals of European origin, but also including a small number of participants from other ethnicities. Therefore, it is not possible to further explore the correlation between intestinal flora and disease through subgroup analysis. It is also not possible to directly generalize the results of the analysis to other racial groups. Secondly, the smallest category of gut flora analyzed in this study was the genus, which limited the exploration of the causal relationship between gut microbiota and disease at the level of specific bacterial species. Thirdly, we chose *p* < 1.0 × 10^–5^ as the screening criterion for gut microbiota in order to obtain sufficient IV. Fourth, Data obtained from public databases usually consist of results from genome-wide association studies, including genotype frequencies, genetic variations, and disease associations. However, they generally lack clinical information. As a result, we are unable to conduct subgroup analyses on important clinical characteristics of ITP patients, such as gender, age, new diagnosis or chronic phase, treatment regimens, prognosis, and mortality. These clinical characteristics are crucial for discussing the relationship between the pathogenesis of ITP and gut microbiota. In future clinical studies, we will make efforts to collect and enhance these essential clinical details to facilitate a more comprehensive discussion on the connection between the pathogenesis of ITP and gut microbiota.

## Conclusion

In summary, the current study indicates a potential cause-and-effect association between the composition of gut microbiota and the development of ITP and HSP, as inferred from the examination of Mendelian randomization involving 196 gut microbiota. The genes predicted that genera Coprococcus3 and genus Gordonibacter were found to be correlated with a heightened susceptibility to ITP. Family Actinomycetaceae, order Actinomycetales was negatively associated with HSP. This study identified specific microbiota associated with ITP, HSP by MR analysis. These findings may offer insights into the pathogenesis of these conditions and propose new therapeutic approaches.

### Supplementary Information

Below is the link to the electronic supplementary material.

Supplementary file1 (DOCX 17 KB)

Supplementary file2 (XLSX 396 KB)

Supplementary file3 (DOCX 33 KB)

Supplementary file4 (TIF 29618 KB)

Supplementary file5 (DOCX 21 KB)

Supplementary file6 (TIF 25403 KB)

Supplementary file7 (DOCX 27 KB)

## Data Availability

The data for this study were sourced from publicly available databases. The data on HSP/ITP/sTP was obtained from the FinnGen release data R9 (https://www.finngen.fi/en/access_results), while the data on the human gut microbiome was acquired from the GWAS dataset of the MiBioGen International Consortium (https://mibiogen.gcc.rug.nl/).
