# Supplementary material for: Mendelian randomization reveals association of gut microbiota with Henoch–Schönlein purpura and immune thrombocytopenia
Source: Int J Hematol. 2024 Apr 26;120(1):50–9. doi: 10.1007/s12185-024-03777-1 (PMC11226487; doi:10.1007/s12185-024-03777-1)
Supplement: Supplementary file 1 — Supplementary file1 (DOCX 17 KB) [file 12185_2024_3777_MOESM1_ESM.docx]

| **ITP** | **HSP** | **sTP** | **反向MR** |
| --- | --- | --- | --- |
| #install packages install.packages("devtools") devtools::install_github("MRCIEU/TwoSampleMR") #install.packages("ggplot2") #library library(TwoSampleMR) library(ggplot2) #set up your work directory setwd("C:\\Users\\81677\\Desktop\\A") #load exposure data expo_rt<- read_exposure_data(  filename = "genus.Bacteroides.txt",  sep = "\t",  snp_col = "rsID",  beta_col = "beta",  se_col = "SE",  effect_allele_col = "eff.allele",  other_allele_col = "re.allele",  pval_col = "P",  samplesize_col = "N" ) #data filter expo_rt<- expo_rt[expo_rt$pval.exposure < 1e-5,] expo_rt <- clump_data(expo_rt,clump_kb = 10000,  clump_r2 = 0.001) write.table(expo_rt, "exposure.txt",row.names = F,sep = "\t",quote = F) #load outcome data outc_rt <- read_outcome_data(  snps = expo_rt$SNP,  filename = "finngen_R9_D3_ITP.txt",  sep = "\t",  snp_col = "rsids",  beta_col = "beta",  se_col = "sebeta",  effect_allele_col = "alt",  other_allele_col = "ref",  eaf_col = "af_alt",  pval_col = "pval") #harmonise and merge data harm_rt <- harmonise_data(  exposure_dat = expo_rt,   outcome_dat = outc_rt,action=1) write.table(harm_rt, "harmonise.txt",row.names = F,sep = "\t",quote = F) #mendelian randomization(MR) analysis mr_result<- mr(harm_rt) View(mr_result) write.table(mr_result, "mr_result.txt",row.names = F,sep = "\t",quote = F) OR=generate_odds_ratios(mr_result) write.table(OR[,5:ncol(OR)],"OR.txt",row.names = F,sep = "\t",quote = F) #heterogeneity test mr_heterogeneity(harm_rt) #outlier test #run_mr_presso(harm_rt,NbDistribution = 1000) #pleiotropy test mr_pleiotropy_test(harm_rt) #obtain the beta for each SNP singlesnp_res<- mr_singlesnp(harm_rt) #View(singlesnp_res) singlesnpOR=generate_odds_ratios(singlesnp_res) write.table(singlesnpOR,"singlesnpOR.txt",row.names = F,sep = "\t",quote = F)  #sensitivity analysis sen_res<- mr_leaveoneout(harm_rt) #View(sen_res) #Scatter plots of several statistical methods p1 <- mr_scatter_plot(mr_result, harm_rt) p1[[1]] ggsave(p1[[1]], file="scatter.pdf", width=8, height=8) #forest plot p2 <- mr_forest_plot(singlesnp_res) p2[[1]] ggsave(p2[[1]], file="forest.pdf", width=8, height=8) #sensitivity analysis plot p3 <- mr_leaveoneout_plot(sen_res) p3[[1]] ggsave(p3[[1]], file="sensitivity analysis.pdf", width=8, height=8) #funnel plot res_single <- mr_singlesnp(harm_rt) p4 <- mr_funnel_plot(singlesnp_res) p4[[1]] ggsave(p4[[1]], file="funnel plot.pdf", width=8, height=8) | #install packages install.packages("devtools") devtools::install_github("MRCIEU/TwoSampleMR")  #install.packages("ggplot2")  #library library(TwoSampleMR) library(ggplot2)    #set up your work directory setwd("C:\\Users\\86177\\Desktop\\A") #load exposure data expo_rt<- read_exposure_data(  filename = "genus.Bifidobacterium2.txt",  sep = "\t",  snp_col = "rsID",  beta_col = "beta",  se_col = "SE",  effect_allele_col = "eff.allele",  other_allele_col = "re.allele",  pval_col = "P",  samplesize_col = "N" ) #data filter expo_rt<- expo_rt[expo_rt$pval.exposure < 1e-5,] expo_rt <- clump_data(expo_rt,clump_kb = 10000,  clump_r2 = 0.001) write.table(expo_rt, "exposure.txt",row.names = F,sep = "\t",quote = F)  #load outcome data outc_rt <- read_outcome_data(snps = expo_rt$SNP,  filename = "finngen_R9_D3_ALLERGPURPURA.txt",  sep = "\t",  snp_col = "rsids",  beta_col = "beta",  se_col = "sebeta",  effect_allele_col = "alt",  other_allele_col = "ref",  eaf_col = "af_alt",  pval_col = "pval")   #harmonise and merge data harm_rt <- harmonise_data(  exposure_dat = expo_rt,   outcome_dat = outc_rt,action=1)   write.table(harm_rt, "harmonise.txt",row.names = F,sep = "\t",quote = F)  #mendelian randomization(MR) analysis mr_result<- mr(harm_rt) View(mr_result) write.table(mr_result, "mr_result.txt",row.names = F,sep = "\t",quote = F)  OR=generate_odds_ratios(mr_result) write.table(OR[,5:ncol(OR)],"OR.txt",row.names = F,sep = "\t",quote = F)   #heterogeneity test mr_heterogeneity(harm_rt)  #outlier test run_mr_presso(harm_rt,NbDistribution = 1000)  #pleiotropy test mr_pleiotropy_test(harm_rt)  #obtain the beta for each SNP singlesnp_res<- mr_singlesnp(harm_rt) View(singlesnp_res) singlesnpOR=generate_odds_ratios(singlesnp_res) write.table(singlesnpOR,"singlesnpOR.txt",row.names = F,sep = "\t",quote = F)  #sensitivity analysis sen_res<- mr_leaveoneout(harm_rt) View(sen_res)  #Scatter plots of several statistical methods p1 <- mr_scatter_plot(my_mr_result, harm_rt) p1[[1]] ggsave(p1[[1]], file="scatter.pdf", width=8, height=8)   #forest plot p2 <- mr_forest_plot(singlesnp_res) p2[[1]] ggsave(p2[[1]], file="forest.pdf", width=8, height=8)   #sensitivity analysis plot p3 <- mr_leaveoneout_plot(sen_res) p3[[1]] ggsave(p3[[1]], file="sensitivity analysis.pdf", width=8, height=8)   #funnel plot res_single <- mr_singlesnp(harm_rt) p4 <- mr_funnel_plot(singlesnp_res) p4[[1]] ggsave(p4[[1]], file="funnel plot.pdf", width=8, height=8) | #install packages install.packages("devtools") devtools::install_github("MRCIEU/TwoSampleMR")  #install.packages("ggplot2")  #library library(TwoSampleMR) library(ggplot2)    #set up your work directory setwd("C:\\Users\\86177I\\Desktop\\A")  #load exposure data expo_rt<- read_exposure_data(  filename = "genus.RuminococcaceaeUCG009.txt",  sep = "\t",  snp_col = "rsID",  beta_col = "beta",  se_col = "SE",  effect_allele_col = "eff.allele",  other_allele_col = "re.allele",  pval_col = "P",  samplesize_col = "N" ) #data filter expo_rt<- expo_rt[expo_rt$pval.exposure < 1e-5,] expo_rt <- clump_data(expo_rt,clump_kb = 10000,  clump_r2 = 0.001) write.table(expo_rt, "exposure.txt",row.names = F,sep = "\t",quote = F)  #load outcome data outc_rt <- read_outcome_data(  snps = expo_rt$SNP,  filename = "finngen_R9_D3_SCNDTHROMBOCYTOPENIA.txt",  sep = "\t",  snp_col = "rsids",  beta_col = "beta",  se_col = "sebeta",  effect_allele_col = "alt",  other_allele_col = "ref",  eaf_col = "af_alt",  pval_col = "pval")   #harmonise and merge data harm_rt <- harmonise_data(  exposure_dat = expo_rt,   outcome_dat = outc_rt,action=1)   write.table(harm_rt, "harmonise.txt",row.names = F,sep = "\t",quote = F)  #mendelian randomization(MR) analysis mr_result<- mr(harm_rt) View(mr_result) write.table(mr_result, "mr_result.txt",row.names = F,sep = "\t",quote = F)  OR=generate_odds_ratios(mr_result) write.table(OR[,5:ncol(OR)],"OR.txt",row.names = F,sep = "\t",quote = F)   #heterogeneity test mr_heterogeneity(harm_rt)  #outlier test run_mr_presso(harm_rt,NbDistribution = 1000)  #pleiotropy test mr_pleiotropy_test(harm_rt)  #obtain the beta for each SNP singlesnp_res<- mr_singlesnp(harm_rt) View(singlesnp_res) singlesnpOR=generate_odds_ratios(singlesnp_res) write.table(singlesnpOR,"singlesnpOR.txt",row.names = F,sep = "\t",quote = F)  #sensitivity analysis sen_res<- mr_leaveoneout(harm_rt) View(sen_res)  #Scatter plots of several statistical methods p1 <- mr_scatter_plot(my_mr_result, harm_rt) p1[[1]] ggsave(p1[[1]], file="scatter.pdf", width=8, height=8)   #forest plot p2 <- mr_forest_plot(singlesnp_res) p2[[1]] ggsave(p2[[1]], file="forest.pdf", width=8, height=8)   #sensitivity analysis plot p3 <- mr_leaveoneout_plot(sen_res) p3[[1]] ggsave(p3[[1]], file="sensitivity analysis.pdf", width=8, height=8)   #funnel plot res_single <- mr_singlesnp(harm_rt) p4 <- mr_funnel_plot(singlesnp_res) p4[[1]] ggsave(p4[[1]], file="funnel plot.pdf", width=8, height=8) | #library library(TwoSampleMR) library(ggplot2)  #set up your work directory setwd("C:\\Users\\86177\\Desktop\\A")  #load exposure data expo_rt<- read_exposure_data(  filename = "finngen_R9_D3_ITP.txt",  sep = "\t",  snp_col = "rsids",  beta_col = "beta",  se_col = "sebeta",  effect_allele_col = "alt",  other_allele_col = "ref",  eaf_col = "af_alt",  pval_col = "pval" ) #data filter expo_rt<- expo_rt[expo_rt$pval.exposure < 1e-5,] expo_rt <- clump_data(expo_rt,clump_kb = 10000,  clump_r2 = 0.001) write.table(expo_rt, "exposure.txt",row.names = F,sep = "\t",quote = F)  #load outcome data outc_rt <- read_outcome_data(filename = "genus..Eubacteriumruminantiumgroup.id.11340.summary.txt",  snps = expo_rt$SNP,  sep = "\t",  snp_col = "rsID",  beta_col = "beta",  se_col = "SE",  effect_allele_col = "eff.allele",  other_allele_col = "re.allele",  pval_col = "P" ) #harmonise and merge data harm_rt <- harmonise_data(  exposure_dat = expo_rt,   outcome_dat = outc_rt,action=1)   write.table(harm_rt, "harmonise.txt",row.names = F,sep = "\t",quote = F)  #mendelian randomization(MR) analysis mr_result<- mr(harm_rt) View(mr_result) write.table(mr_result, "mr_result.txt",row.names = F,sep = "\t",quote = F) OR=generate_odds_ratios(mr_result) write.table(OR[,5:ncol(OR)],"OR.txt",row.names = F,sep = "\t",quote = F)   #heterogeneity test mr_heterogeneity(harm_rt)  #outlier test #run_mr_presso(harm_rt,NbDistribution = 1000)  #pleiotropy test mr_pleiotropy_test(harm_rt)  #obtain the beta for each SNP singlesnp_res<- mr_singlesnp(harm_rt) #View(singlesnp_res) singlesnpOR=generate_odds_ratios(singlesnp_res) write.table(singlesnpOR,"singlesnpOR.txt",row.names = F,sep = "\t",quote = F)  #sensitivity analysis sen_res<- mr_leaveoneout(harm_rt) #View(sen_res)  #Scatter plots of several statistical methods p1 <- mr_scatter_plot(mr_result, harm_rt) p1[[1]] ggsave(p1[[1]], file="scatter.pdf", width=8, height=8)   #forest plot p2 <- mr_forest_plot(singlesnp_res) p2[[1]] ggsave(p2[[1]], file="forest.pdf", width=8, height=8)   #sensitivity analysis plot p3 <- mr_leaveoneout_plot(sen_res) p3[[1]] ggsave(p3[[1]], file="sensitivity analysis.pdf", width=8, height=8)   #funnel plot res_single <- mr_singlesnp(harm_rt) p4 <- mr_funnel_plot(singlesnp_res) p4[[1]] ggsave(p4[[1]], file="funnel plot.pdf", width=8, height=8) |
