# Supplementary material for: Mendelian randomization reveals association of gut microbiota with Henoch–Schönlein purpura and immune thrombocytopenia
Source: Int J Hematol. 2024 Apr 26;120(1):50–9. doi: 10.1007/s12185-024-03777-1 (PMC11226487; doi:10.1007/s12185-024-03777-1)
Supplement: Supplementary file 3 — Supplementary file3 (DOCX 33 KB) [file 12185_2024_3777_MOESM3_ESM.docx]

| **Gut microbiota** | **SNP** | **Other**  **allele** | **Effect**  **allele** | **beta** | **SE** | **P value** | **Sample size** |
| --- | --- | --- | --- | --- | --- | --- | --- |
| class.Methanobacteria | rs73457410 | G | A | 0.21534 | 0.04367 | 1.41E-06 | 3498 |
| class.Methanobacteria | rs10202904 | G | T | -0.12175 | 0.023536 | 3.01E-07 | 3695 |
| class.Methanobacteria | rs62241835 | T | G | -0.20339 | 0.041793 | 1.63E-06 | 3383 |
| class.Methanobacteria | rs894996 | A | C | 0.216999 | 0.044908 | 1.88E-06 | 3582 |
| class.Methanobacteria | rs11018665 | T | A | 0.111465 | 0.025052 | 6.52E-06 | 3698 |
| class.Methanobacteria | rs75208022 | T | C | -0.22724 | 0.048763 | 5.92E-06 | 3203 |
| class.Methanobacteria | rs12825290 | G | C | -0.21678 | 0.04935 | 6.08E-06 | 3144 |
| class.Methanobacteria | rs56131665 | A | G | 0.178661 | 0.039298 | 6.18E-06 | 3572 |
| class.Methanobacteria | rs4257531 | A | G | 0.164498 | 0.036496 | 7.44E-06 | 3162 |
| class.Methanobacteria | rs10424197 | A | G | -0.11127 | 0.024752 | 9.28E-06 | 3698 |
| class.Methanobacteria | rs6508769 | C | T | 0.153519 | 0.034452 | 8.23E-06 | 3319 |
| class.Methanobacteria | rs73068003 | T | G | -0.15814 | 0.03518 | 8.45E-06 | 3692 |
| family.BacteroidalesS24 | rs738193 | C | T | 0.084733 | 0.016586 | 3.82E-07 | 7778 |
| family.BacteroidalesS25 | rs941000 | T | C | 0.085027 | 0.016338 | 3.16E-07 | 7778 |
| family.BacteroidalesS26 | rs689695 | A | C | 0.081458 | 0.016706 | 1.28E-06 | 7954 |
| family.BacteroidalesS27 | rs17043785 | C | T | -0.17619 | 0.034713 | 5.12E-07 | 5208 |
| family.BacteroidalesS28 | rs10872669 | G | A | -0.12307 | 0.027567 | 9.49E-06 | 7368 |
| family.BacteroidalesS29 | rs12748533 | T | G | -0.08211 | 0.01727 | 2.59E-06 | 7948 |
| family.BacteroidalesS30 | rs6831034 | A | T | -0.09565 | 0.020523 | 6.10E-06 | 7576 |
| family.BacteroidalesS31 | rs61508842 | C | T | 0.122574 | 0.027239 | 7.83E-06 | 7576 |
| family.BacteroidalesS32 | rs78609301 | G | A | -0.08671 | 0.019571 | 7.09E-06 | 7953 |
| family.BacteroidalesS33 | rs7217209 | T | C | 0.084302 | 0.018731 | 8.43E-06 | 7745 |
| family.BacteroidalesS34 | rs11135366 | G | C | 0.084214 | 0.018374 | 8.78E-06 | 7953 |
| family.Lachnospiraceae | rs12760724 | C | A | -0.04846 | 0.010793 | 7.27E-06 | 18340 |
| family.Lachnospiraceae | rs3127230 | T | C | -0.05035 | 0.01122 | 6.20E-06 | 18340 |
| family.Lachnospiraceae | rs9929145 | A | G | -0.12572 | 0.024523 | 2.84E-07 | 15866 |
| family.Lachnospiraceae | rs11755180 | G | C | -0.04923 | 0.010489 | 2.69E-06 | 18333 |
| family.Lachnospiraceae | rs35524804 | C | T | -0.06075 | 0.012522 | 2.45E-06 | 17859 |
| family.Lachnospiraceae | rs11979110 | C | T | -0.05009 | 0.010507 | 1.82E-06 | 18339 |
| family.Lachnospiraceae | rs79086868 | C | T | 0.077672 | 0.016447 | 3.01E-06 | 17522 |
| family.Lachnospiraceae | rs112040820 | G | A | 0.054958 | 0.011709 | 2.42E-06 | 17512 |
| family.Lachnospiraceae | rs2159863 | G | A | -0.05858 | 0.012874 | 3.70E-06 | 18340 |
| family.Lachnospiraceae | rs11139361 | C | T | 0.049446 | 0.01105 | 4.26E-06 | 18333 |
| family.Lachnospiraceae | rs7359994 | C | T | -0.05031 | 0.011248 | 5.36E-06 | 18340 |
| family.Lachnospiraceae | rs1205443 | G | A | 0.050142 | 0.011216 | 7.29E-06 | 17779 |
| family.Lachnospiraceae | rs959845 | T | C | -0.04937 | 0.01078 | 5.17E-06 | 17859 |
| family.Lachnospiraceae | rs146660815 | C | T | -0.21511 | 0.047678 | 6.53E-06 | 4112 |
| family.Lachnospiraceae | rs10402491 | T | C | 0.06617 | 0.014851 | 7.58E-06 | 18334 |
| family.Lachnospiraceae | rs13005175 | G | A | 0.099348 | 0.02184 | 8.37E-06 | 16893 |
| family.Lachnospiraceae | rs2910921 | C | T | 0.160302 | 0.035832 | 8.42E-06 | 6549 |
| family.Lachnospiraceae | rs11841382 | T | G | -0.07181 | 0.017462 | 9.58E-06 | 18340 |
| family.Methanobacteriaceae | rs12825290 | G | C | -0.21678 | 0.04935 | 6.08E-06 | 3144 |
| family.Methanobacteriaceae | rs56131665 | A | G | 0.178661 | 0.039298 | 6.18E-06 | 3572 |
| family.Methanobacteriaceae | rs11018665 | T | A | 0.111465 | 0.025052 | 6.52E-06 | 3698 |
| family.Methanobacteriaceae | rs4257531 | A | G | 0.164498 | 0.036496 | 7.44E-06 | 3162 |
| family.Methanobacteriaceae | rs10424197 | A | G | -0.11127 | 0.024752 | 9.28E-06 | 3698 |
| family.Methanobacteriaceae | rs6508769 | C | T | 0.153519 | 0.034452 | 8.23E-06 | 3319 |
| family.Methanobacteriaceae | rs73068003 | T | G | -0.15814 | 0.03518 | 8.45E-06 | 3692 |
| family.Methanobacteriaceae | rs10202904 | G | T | -0.12175 | 0.023536 | 3.01E-07 | 3695 |
| family.Methanobacteriaceae | rs73457410 | G | A | 0.21534 | 0.04367 | 1.41E-06 | 3498 |
| family.Methanobacteriaceae | rs62241835 | T | G | -0.20339 | 0.041793 | 1.63E-06 | 3383 |
| family.Methanobacteriaceae | rs894996 | A | C | 0.216999 | 0.044908 | 1.88E-06 | 3582 |
| family.Methanobacteriaceae | rs75208022 | T | C | -0.22724 | 0.048763 | 5.92E-06 | 3203 |
| genus..Eubacteriumhalliigroup | rs10798999 | T | C | 0.060166 | 0.012675 | 2.61E-06 | 16561 |
| genus..Eubacteriumhalliigroup | rs13116360 | C | T | 0.154124 | 0.029719 | 2.94E-07 | 10323 |
| genus..Eubacteriumhalliigroup | rs949971 | G | T | -0.05402 | 0.01161 | 3.29E-06 | 16566 |
| genus..Eubacteriumhalliigroup | rs60254196 | G | A | -0.05228 | 0.011186 | 2.70E-06 | 16238 |
| genus..Eubacteriumhalliigroup | rs10808115 | C | A | -0.05047 | 0.010992 | 4.42E-06 | 16566 |
| genus..Eubacteriumhalliigroup | rs10501370 | T | C | -0.11559 | 0.025249 | 5.42E-06 | 13824 |
| genus..Eubacteriumhalliigroup | rs74018587 | T | C | 0.208943 | 0.043822 | 3.70E-06 | 3039 |
| genus..Eubacteriumhalliigroup | rs6550770 | C | T | -0.19809 | 0.044354 | 4.82E-06 | 4279 |
| genus..Eubacteriumhalliigroup | rs138531890 | G | A | 0.153138 | 0.034946 | 5.43E-06 | 7710 |
| genus..Eubacteriumhalliigroup | rs281379 | G | A | -0.04995 | 0.011215 | 9.33E-06 | 16130 |
| genus..Eubacteriumhalliigroup | rs78056098 | T | G | -0.05074 | 0.011376 | 8.29E-06 | 16568 |
| genus..Eubacteriumhalliigroup | rs28584818 | G | A | 0.126115 | 0.026863 | 4.43E-06 | 11095 |
| genus..Eubacteriumhalliigroup | rs117748144 | C | T | -0.12658 | 0.028712 | 7.86E-06 | 10861 |
| genus..Eubacteriumhalliigroup | rs17474256 | A | G | 0.081081 | 0.018457 | 9.45E-06 | 16460 |
| genus..Eubacteriumhalliigroup | rs630939 | T | C | -0.05089 | 0.011435 | 9.16E-06 | 16238 |
| genus..Eubacteriumhalliigroup | rs17074066 | C | T | -0.0814 | 0.018927 | 9.35E-06 | 15804 |
| genus..Eubacteriumruminantiumgroup | rs10131724 | A | C | -0.19983 | 0.041458 | 2.39E-06 | 5071 |
| genus..Eubacteriumruminantiumgroup | rs10923018 | G | A | 0.072644 | 0.016092 | 6.80E-06 | 7739 |
| genus..Eubacteriumruminantiumgroup | rs112375806 | T | A | 0.143141 | 0.029376 | 5.82E-06 | 7123 |
| genus..Eubacteriumruminantiumgroup | rs11637981 | G | T | -0.07326 | 0.016089 | 5.44E-06 | 7718 |
| genus..Eubacteriumruminantiumgroup | rs13025464 | T | C | -0.07371 | 0.016379 | 6.97E-06 | 7738 |
| genus..Eubacteriumruminantiumgroup | rs139749 | C | T | -0.08454 | 0.017179 | 8.59E-07 | 7314 |
| genus..Eubacteriumruminantiumgroup | rs16891896 | G | A | -0.17479 | 0.039057 | 2.38E-06 | 4753 |
| genus..Eubacteriumruminantiumgroup | rs17519472 | C | T | 0.107804 | 0.023398 | 4.70E-06 | 7710 |
| genus..Eubacteriumruminantiumgroup | rs209813 | G | A | -0.10349 | 0.023639 | 9.23E-06 | 7264 |
| genus..Eubacteriumruminantiumgroup | rs2116427 | A | G | 0.091146 | 0.018235 | 4.67E-07 | 7739 |
| genus..Eubacteriumruminantiumgroup | rs2229917 | A | G | 0.153538 | 0.032392 | 2.16E-06 | 7050 |
| genus..Eubacteriumruminantiumgroup | rs2418654 | C | T | -0.07489 | 0.016585 | 6.17E-06 | 7314 |
| genus..Eubacteriumruminantiumgroup | rs2817174 | C | T | -0.07343 | 0.016369 | 7.87E-06 | 7710 |
| genus..Eubacteriumruminantiumgroup | rs57340348 | T | C | -0.09794 | 0.021217 | 4.93E-06 | 7314 |
| genus..Eubacteriumruminantiumgroup | rs606117 | A | G | 0.083324 | 0.018056 | 4.82E-06 | 7263 |
| genus..Eubacteriumruminantiumgroup | rs6676699 | G | T | -0.08881 | 0.019645 | 6.38E-06 | 7708 |
| genus..Eubacteriumruminantiumgroup | rs7000472 | A | G | -0.07623 | 0.016523 | 4.07E-06 | 7739 |
| genus..Eubacteriumruminantiumgroup | rs72836424 | C | T | -0.13982 | 0.030069 | 2.62E-06 | 7314 |
| genus..Eubacteriumruminantiumgroup | rs73139629 | A | C | -0.1151 | 0.024791 | 5.36E-06 | 7314 |
| genus.Allisonella | rs602075 | G | A | 0.168974 | 0.029698 | 3.57E-08 | 3212 |
| genus.Allisonella | rs6742198 | A | G | 0.149152 | 0.031648 | 3.35E-06 | 3182 |
| genus.Allisonella | rs35778461 | T | C | 0.146679 | 0.029719 | 1.21E-06 | 3212 |
| genus.Allisonella | rs1901739 | G | T | 0.115769 | 0.024863 | 3.59E-06 | 3212 |
| genus.Allisonella | rs76904847 | A | G | 0.148523 | 0.033486 | 6.09E-06 | 3182 |
| genus.Allisonella | rs35110698 | C | T | -0.14632 | 0.032085 | 5.72E-06 | 3212 |
| genus.Allisonella | rs7898615 | G | T | 0.167966 | 0.037359 | 8.87E-06 | 3212 |
| genus.Allisonella | rs594561 | T | C | 0.112231 | 0.025168 | 9.41E-06 | 3212 |
| genus.Allisonella | rs685403 | C | G | -0.1752 | 0.040447 | 4.88E-06 | 3212 |
| genus.Coprococcus2 | rs12634070 | C | T | 0.073649 | 0.016492 | 9.95E-06 | 11457 |
| genus.Coprococcus3 | rs61823518 | C | A | -0.09554 | 0.021572 | 6.68E-06 | 10980 |
| genus.Coprococcus4 | rs10070053 | G | A | 0.059431 | 0.013538 | 7.65E-06 | 11473 |
| genus.Coprococcus5 | rs2482516 | T | C | 0.075441 | 0.016462 | 4.72E-06 | 11483 |
| genus.Coprococcus6 | rs59936925 | T | A | 0.117085 | 0.023399 | 9.38E-07 | 10931 |
| genus.Coprococcus7 | rs6677933 | T | C | -0.08044 | 0.016422 | 1.19E-06 | 11483 |
| genus.Coprococcus8 | rs1958519 | A | T | 0.066529 | 0.013856 | 1.58E-06 | 11009 |
| genus.Coprococcus9 | rs72680320 | C | T | -0.06494 | 0.013919 | 2.27E-06 | 11009 |
| genus.Coprococcus10 | rs35890118 | G | A | -0.06654 | 0.014766 | 8.26E-06 | 11478 |
| genus.Coprococcus11 | rs9426473 | G | A | 0.072736 | 0.016166 | 6.31E-06 | 11486 |
| genus.Coprococcus3 | rs8100692 | C | T | 0.057741 | 0.011348 | 4.16E-07 | 15829 |
| genus.Coprococcus4 | rs62481985 | C | G | -0.05824 | 0.011505 | 4.18E-07 | 15472 |
| genus.Coprococcus5 | rs178271 | C | T | 0.145257 | 0.029424 | 7.81E-07 | 10794 |
| genus.Coprococcus6 | rs13394391 | T | C | -0.07093 | 0.015092 | 2.20E-06 | 15834 |
| genus.Coprococcus7 | rs11080344 | T | C | 0.051692 | 0.011305 | 4.79E-06 | 15835 |
| genus.Coprococcus8 | rs7521171 | A | G | -0.05964 | 0.012928 | 4.32E-06 | 15472 |
| genus.Coprococcus9 | rs11077359 | C | T | -0.06454 | 0.014883 | 9.64E-06 | 15378 |
| genus.Coprococcus10 | rs13247359 | A | G | 0.051211 | 0.011303 | 7.33E-06 | 15833 |
| genus.Coprococcus11 | rs4575475 | A | G | 0.061951 | 0.013779 | 7.04E-06 | 15154 |
| genus.Coprococcus12 | rs10810043 | G | A | 0.051552 | 0.011592 | 9.27E-06 | 15833 |
| genus.Gordonibacter | rs76287110 | T | A | -0.24296 | 0.046618 | 1.67E-07 | 3483 |
| genus.Gordonibacter | rs7294633 | T | C | 0.12867 | 0.024995 | 3.44E-07 | 3723 |
| genus.Gordonibacter | rs7220558 | T | A | 0.116884 | 0.023483 | 6.71E-07 | 3723 |
| genus.Gordonibacter | rs72714787 | A | C | 0.181405 | 0.03771 | 1.43E-06 | 3723 |
| genus.Gordonibacter | rs71545975 | G | A | -0.15397 | 0.033892 | 7.04E-06 | 3556 |
| genus.Gordonibacter | rs35042269 | A | C | -0.18029 | 0.04033 | 8.11E-06 | 3595 |
| genus.Gordonibacter | rs322296 | A | G | 0.17869 | 0.037722 | 4.02E-06 | 3595 |
| genus.Gordonibacter | rs72939513 | G | A | -0.21399 | 0.049059 | 7.98E-06 | 3435 |
| genus.Gordonibacter | rs3765837 | G | T | -0.19073 | 0.043361 | 7.17E-06 | 3556 |
| genus.Gordonibacter | rs13412653 | C | A | 0.107597 | 0.023923 | 8.61E-06 | 3723 |
| genus.Gordonibacter | rs117347059 | C | G | -0.12826 | 0.028512 | 9.17E-06 | 3684 |
| genus.Gordonibacter | rs16955299 | A | G | -0.19642 | 0.043354 | 6.37E-06 | 3572 |
| genus.Gordonibacter | rs4596722 | G | A | 0.102908 | 0.023157 | 9.06E-06 | 3723 |
| genus.Gordonibacter | rs768830 | A | G | 0.149852 | 0.033331 | 7.76E-06 | 3723 |
| genus.Gordonibacter | rs61934597 | T | C | -0.17249 | 0.038857 | 8.37E-06 | 3684 |
| order.Bacillales | rs4617108 | G | A | 0.248861 | 0.052593 | 1.98E-06 | 3018 |
| order.Bacillales | rs74887130 | G | C | -0.19217 | 0.035882 | 1.16E-07 | 3218 |
| order.Bacillales | rs62640857 | G | A | 0.148238 | 0.032646 | 4.49E-06 | 3235 |
| order.Bacillales | rs1287018 | A | G | 0.141125 | 0.031931 | 9.87E-06 | 3207 |
| order.Bacillales | rs10410917 | C | T | 0.11471 | 0.025003 | 5.57E-06 | 3239 |
| order.Bacillales | rs74420793 | G | A | -0.16441 | 0.035376 | 3.07E-06 | 3077 |
| order.Bacillales | rs11034576 | G | A | 0.205765 | 0.045331 | 8.86E-06 | 3076 |
| order.Bacillales | rs10233278 | C | T | -0.11632 | 0.024853 | 3.51E-06 | 3239 |
| order.Bacillales | rs11207728 | A | G | -0.14455 | 0.031691 | 5.73E-06 | 3239 |
| order.Bacillales | rs11844714 | G | A | -0.14325 | 0.032027 | 5.06E-06 | 3239 |
| order.Bacillales | rs12522021 | A | T | 0.174339 | 0.040052 | 7.60E-06 | 3235 |
| order.Methanobacteriales | rs10202904 | G | T | -0.12175 | 0.023536 | 3.01E-07 | 3695 |
| order.Methanobacteriales | rs894996 | A | C | 0.216999 | 0.044908 | 1.88E-06 | 3582 |
| order.Methanobacteriales | rs73457410 | G | A | 0.21534 | 0.04367 | 1.41E-06 | 3498 |
| order.Methanobacteriales | rs62241835 | T | G | -0.20339 | 0.041793 | 1.63E-06 | 3383 |
| order.Methanobacteriales | rs4257531 | A | G | 0.164498 | 0.036496 | 7.44E-06 | 3162 |
| order.Methanobacteriales | rs75208022 | T | C | -0.22724 | 0.048763 | 5.92E-06 | 3203 |
| order.Methanobacteriales | rs12825290 | G | C | -0.21678 | 0.04935 | 6.08E-06 | 3144 |
| order.Methanobacteriales | rs56131665 | A | G | 0.178661 | 0.039298 | 6.18E-06 | 3572 |
| order.Methanobacteriales | rs11018665 | T | A | 0.111465 | 0.025052 | 6.52E-06 | 3698 |
| order.Methanobacteriales | rs6508769 | C | T | 0.153519 | 0.034452 | 8.23E-06 | 3319 |
| order.Methanobacteriales | rs73068003 | T | G | -0.15814 | 0.03518 | 8.45E-06 | 3692 |
| order.Methanobacteriales | rs10424197 | A | G | -0.11127 | 0.024752 | 9.28E-06 | 3698 |
