# Supplementary figures and images for: Mendelian randomization reveals association of gut microbiota with Henoch–Schönlein purpura and immune thrombocytopenia
Source: Int J Hematol. 2024 Apr 26;120(1):50–9. doi: 10.1007/s12185-024-03777-1 (PMC11226487; doi:10.1007/s12185-024-03777-1)

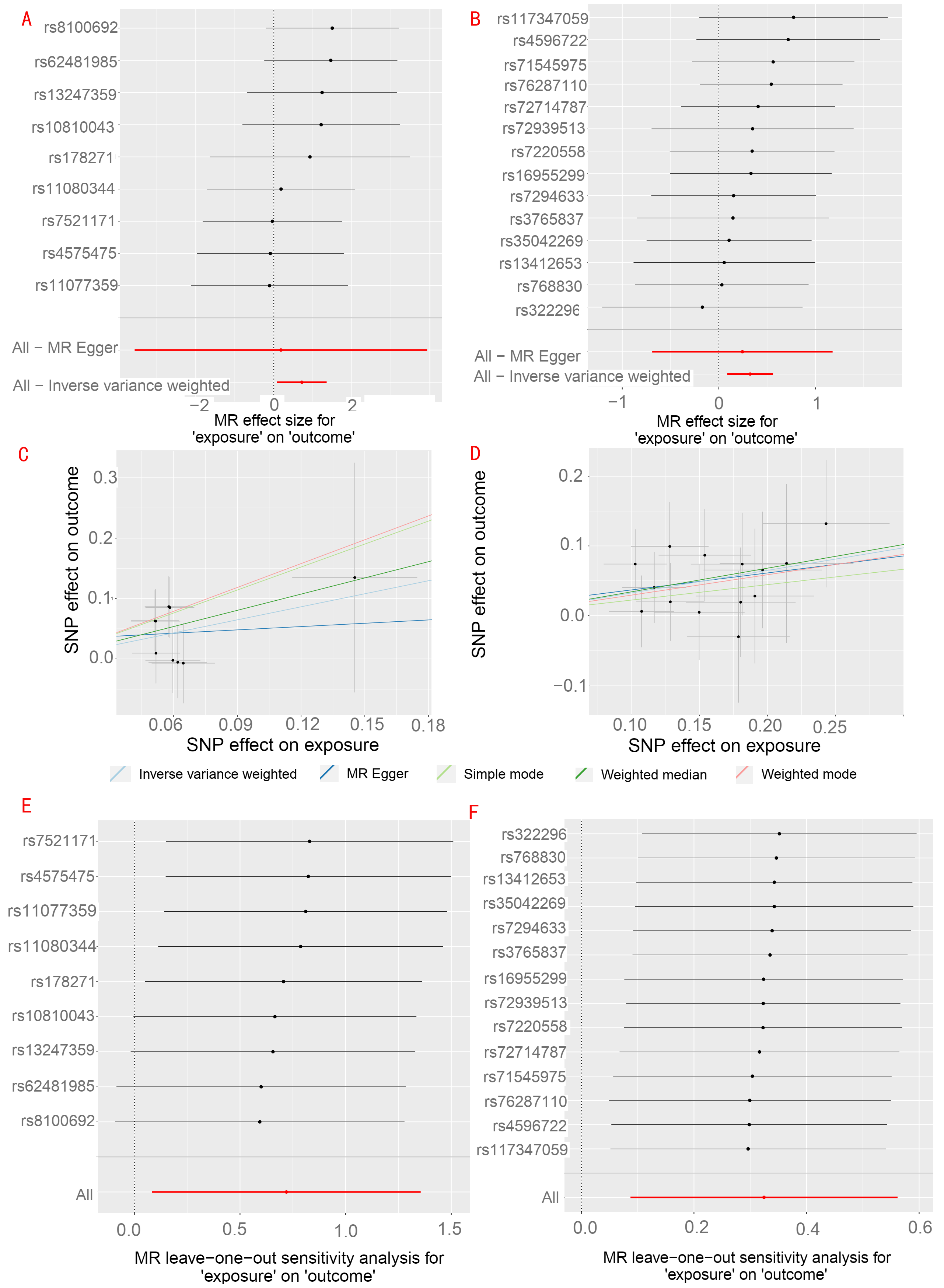

Supplement: Supplementary file 4 — Supplementary file4 (TIF 29618 KB) [file 12185_2024_3777_MOESM4_ESM.tif]

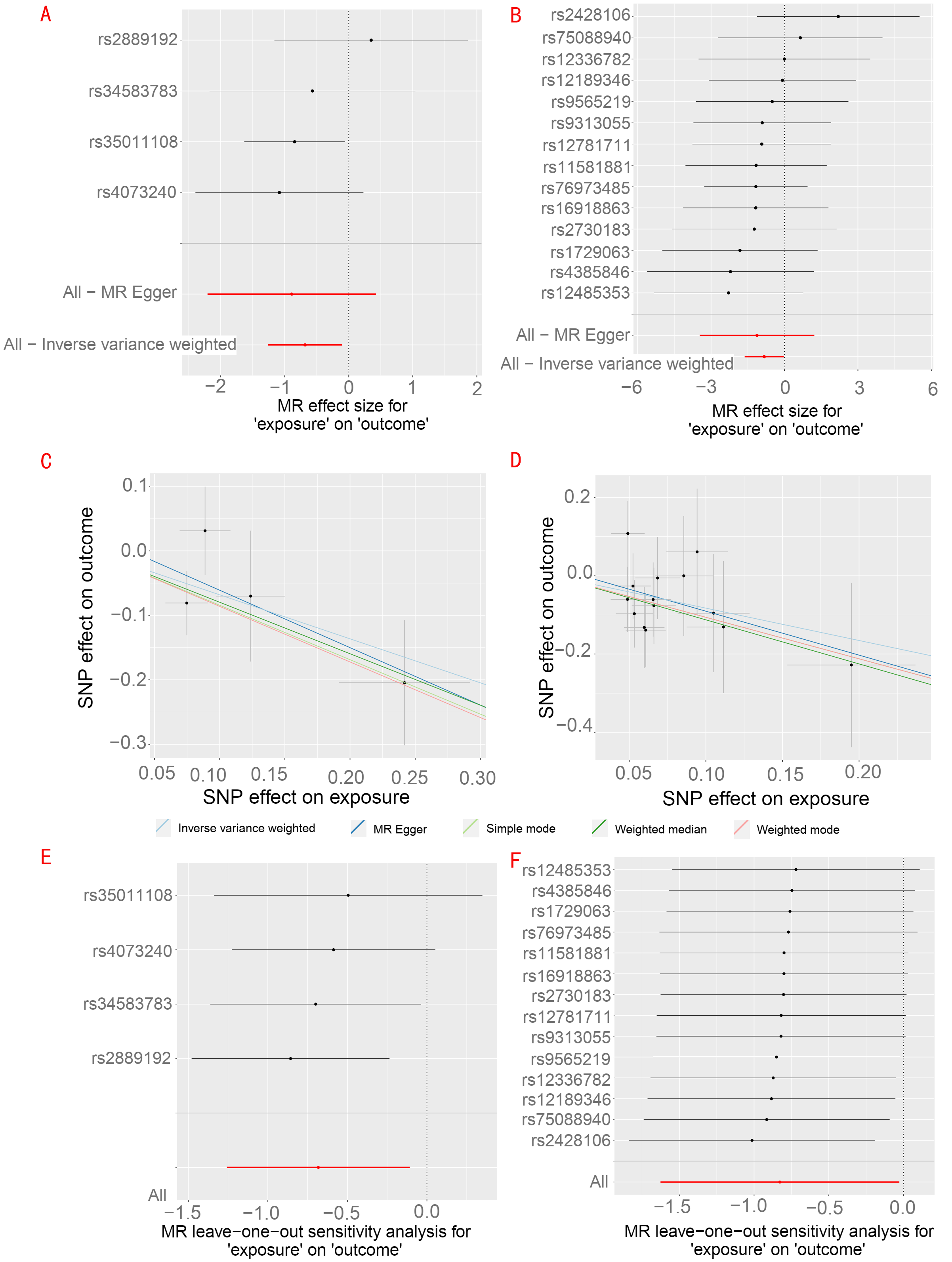

Supplement: Supplementary file 6 — Supplementary file6 (TIF 25403 KB) [file 12185_2024_3777_MOESM6_ESM.tif]
