# Supplementary material for: Mendelian randomization reveals association of gut microbiota with Henoch–Schönlein purpura and immune thrombocytopenia
Source: Int J Hematol. 2024 Apr 26;120(1):50–9. doi: 10.1007/s12185-024-03777-1 (PMC11226487; doi:10.1007/s12185-024-03777-1)
Supplement: Supplementary file 5 — Supplementary file5 (DOCX 21 KB) [file 12185_2024_3777_MOESM5_ESM.docx]

| **Gut microbiota** | **SNP** | **Other**  **allele** | **effect**  **allele** | **beta** | **SE** | **P value** | **Sample Size** |
| --- | --- | --- | --- | --- | --- | --- | --- |
| family.Actinomycetaceae | rs35011108 | G | A | 0.241826 | 0.05038 | 1.83E-06 | 3630 |
| family.Actinomycetaceae | rs2889192 | T | G | 0.088754 | 0.019501 | 3.64E-06 | 7710 |
| family.Actinomycetaceae | rs34583783 | T | G | 0.123756 | 0.026427 | 5.48E-06 | 7710 |
| family.Actinomycetaceae | rs58484246 | C | T | 0.076141 | 0.016901 | 6.12E-06 | 7710 |
| family.Actinomycetaceae | rs4073240 | A | G | 0.074755 | 0.016468 | 6.05E-06 | 7710 |
| genus.Bilophila | rs116261629 | C | G | 0.128112 | 0.026136 | 8.62E-07 | 11276 |
| genus.Bilophila | rs6793291 | A | C | 0.112729 | 0.024163 | 3.11E-06 | 11051 |
| genus.Bilophila | rs1571225 | T | C | 0.082683 | 0.017062 | 1.12E-06 | 12854 |
| genus.Bilophila | rs3827020 | T | C | 0.076648 | 0.016064 | 1.79E-06 | 12628 |
| genus.Bilophila | rs1241171 | A | G | -0.06927 | 0.015015 | 4.24E-06 | 12856 |
| genus.Bilophila | rs7802841 | A | C | 0.06701 | 0.01377 | 1.77E-06 | 12856 |
| genus.Bilophila | rs8013541 | T | A | -0.0572 | 0.012567 | 5.48E-06 | 12856 |
| genus.Bilophila | rs72676854 | C | T | 0.123209 | 0.026882 | 5.62E-06 | 11616 |
| genus.Bilophila | rs1917709 | T | A | 0.118509 | 0.026742 | 7.37E-06 | 10886 |
| genus.Bilophila | rs542415 | C | T | -0.06136 | 0.013344 | 4.71E-06 | 12777 |
| genus.Bilophila | rs4798126 | A | G | 0.073288 | 0.016799 | 7.15E-06 | 12777 |
| genus.Bilophila | rs1969927 | A | G | 0.056459 | 0.012693 | 9.07E-06 | 12777 |
| genus.Bilophila | rs60178956 | A | G | -0.06248 | 0.014148 | 8.06E-06 | 12777 |
| genus.Bilophila | rs2728491 | T | G | -0.06274 | 0.013946 | 6.33E-06 | 12856 |
| genus.Bilophila | rs2713349 | T | A | 0.061723 | 0.014017 | 8.63E-06 | 12853 |
| genus.Bilophila | rs9899990 | G | A | -0.1027 | 0.023388 | 9.07E-06 | 12088 |
| genus.Bilophila | rs11069458 | C | T | -0.06809 | 0.015503 | 7.72E-06 | 12856 |
| genus.FamilyXIIIAD3011group | rs62200412 | T | C | -0.08009 | 0.016383 | 5.80E-07 | 13661 |
| genus.FamilyXIIIAD3012group | rs9852893 | G | C | 0.065783 | 0.012906 | 3.88E-07 | 14184 |
| genus.FamilyXIIIAD3013group | rs17156849 | A | G | -0.11289 | 0.024529 | 4.19E-06 | 13352 |
| genus.FamilyXIIIAD3014group | rs16840310 | G | A | -0.06081 | 0.012215 | 6.75E-07 | 14201 |
| genus.FamilyXIIIAD3015group | rs72730932 | A | C | -0.08996 | 0.017711 | 6.89E-07 | 13102 |
| genus.FamilyXIIIAD3016group | rs16940167 | T | C | 0.073255 | 0.01599 | 3.91E-06 | 14201 |
| genus.FamilyXIIIAD3017group | rs62029761 | G | A | 0.128753 | 0.0276 | 3.89E-06 | 9655 |
| genus.FamilyXIIIAD3018group | rs11736617 | A | G | -0.07595 | 0.01721 | 9.02E-06 | 13778 |
| genus.FamilyXIIIAD3019group | rs12812672 | C | T | -0.09607 | 0.020828 | 2.56E-06 | 13565 |
| genus.FamilyXIIIAD3020group | rs9276029 | G | A | -0.08114 | 0.018567 | 8.93E-06 | 13566 |
| genus.FamilyXIIIAD3021group | rs12911842 | T | A | -0.0812 | 0.018334 | 6.91E-06 | 14125 |
| genus.FamilyXIIIAD3022group | rs149302 | C | T | -0.06456 | 0.014322 | 7.48E-06 | 14192 |
| genus.FamilyXIIIAD3023group | rs9837139 | G | A | 0.10752 | 0.024048 | 8.71E-06 | 13527 |
| genus.FamilyXIIIAD3024group | rs739451 | T | C | 0.064959 | 0.014753 | 7.88E-06 | 14197 |
| genus.FamilyXIIIAD3025group | rs11126423 | T | C | 0.09044 | 0.01963 | 5.91E-06 | 13735 |
| genus.Marvinbryantia | rs61884471 | A | G | 0.124426 | 0.024843 | 1.01E-06 | 11559 |
| genus.Marvinbryantia | rs2724813 | G | A | -0.08408 | 0.016755 | 6.28E-07 | 11610 |
| genus.Marvinbryantia | rs1187983 | T | C | -0.09355 | 0.019317 | 2.02E-06 | 11896 |
| genus.Marvinbryantia | rs2842896 | T | C | -0.06494 | 0.013115 | 7.25E-07 | 11709 |
| genus.Marvinbryantia | rs11645029 | C | G | -0.06062 | 0.013155 | 4.15E-06 | 11709 |
| genus.Marvinbryantia | rs12963345 | C | G | -0.05968 | 0.013229 | 6.60E-06 | 11709 |
| genus.Marvinbryantia | rs2863363 | G | A | 0.063486 | 0.013632 | 3.11E-06 | 11950 |
| genus.Marvinbryantia | rs72948274 | C | A | -0.12635 | 0.027221 | 3.26E-06 | 11561 |
| genus.Marvinbryantia | rs146541147 | A | G | 0.118845 | 0.026842 | 6.86E-06 | 10778 |
| genus.Marvinbryantia | rs11620597 | C | T | 0.119479 | 0.027169 | 7.80E-06 | 11031 |
| genus.Marvinbryantia | rs8006832 | T | G | -0.09524 | 0.02167 | 6.58E-06 | 11412 |
| genus.Marvinbryantia | rs3125832 | C | A | 0.067932 | 0.015012 | 5.03E-06 | 11709 |
| genus.Roseburia | rs2160994 | C | T | 0.055069 | 0.011248 | 9.70E-07 | 17444 |
| genus.Roseburia | rs2034589 | C | G | 0.062976 | 0.012349 | 5.01E-07 | 17846 |
| genus.Roseburia | rs116270582 | A | T | -0.15384 | 0.032932 | 1.20E-06 | 9099 |
| genus.Roseburia | rs16910295 | C | T | -0.09804 | 0.020957 | 2.91E-06 | 16648 |
| genus.Roseburia | rs12740451 | C | T | 0.069753 | 0.015361 | 7.34E-06 | 17837 |
| genus.Roseburia | rs6445851 | A | G | -0.04973 | 0.010816 | 3.53E-06 | 17851 |
| genus.Roseburia | rs9300744 | T | C | -0.05885 | 0.012623 | 4.75E-06 | 17854 |
| genus.Roseburia | rs2943022 | C | T | 0.049379 | 0.010676 | 4.11E-06 | 17854 |
| genus.Roseburia | rs6930661 | T | C | -0.09616 | 0.020497 | 2.48E-06 | 16945 |
| genus.Roseburia | rs4748237 | C | G | 0.048833 | 0.01064 | 4.67E-06 | 17851 |
| genus.Roseburia | rs147990086 | G | A | -0.05789 | 0.01324 | 8.93E-06 | 17840 |
| genus.Roseburia | rs329182 | C | T | 0.069033 | 0.015288 | 5.90E-06 | 17854 |
| genus.Roseburia | rs302266 | C | T | -0.07773 | 0.017299 | 8.13E-06 | 17056 |
| genus.Roseburia | rs75326254 | T | C | -0.10463 | 0.02309 | 7.50E-06 | 15893 |
| genus.Roseburia | rs55858165 | C | A | 0.179284 | 0.040495 | 9.99E-06 | 5447 |
| genus.Roseburia | rs57466170 | T | C | 0.074141 | 0.01716 | 8.30E-06 | 17333 |
| genus.Roseburia | rs78753150 | C | A | 0.096874 | 0.021407 | 9.98E-06 | 16303 |
| order.Actinomycetales | rs35011108 | G | A | 0.241546 | 0.05038 | 1.88E-06 | 3630 |
| order.Actinomycetales | rs2889192 | T | G | 0.088405 | 0.0195 | 3.97E-06 | 7711 |
| order.Actinomycetales | rs34583783 | T | G | 0.123688 | 0.026425 | 5.54E-06 | 7711 |
| order.Actinomycetales | rs4073240 | A | G | 0.074973 | 0.016467 | 5.68E-06 | 7711 |
| order.Actinomycetales | rs58484246 | C | T | 0.075828 | 0.0169 | 6.67E-06 | 7711 |
