# Supplementary material for: Mendelian randomization reveals association of gut microbiota with Henoch–Schönlein purpura and immune thrombocytopenia
Source: Int J Hematol. 2024 Apr 26;120(1):50–9. doi: 10.1007/s12185-024-03777-1 (PMC11226487; doi:10.1007/s12185-024-03777-1)
Supplement: Supplementary file 7 — Supplementary file7 (DOCX 27 KB) [file 12185_2024_3777_MOESM7_ESM.docx]

| **Gut microbiota** | **SNP** | **Other**  **allele** | **Effect**  **allele** | **beta** | **SE** | **P value** | **Sample size** |
| --- | --- | --- | --- | --- | --- | --- | --- |
| class.Methanobacteria | rs73457410 | G | A | 0.215 | 0.04367 | 1.41E-06 | 3498 |
| class.Methanobacteria | rs10202904 | G | T | -0.12 | 0.023536 | 3.01E-07 | 3695 |
| class.Methanobacteria | rs62241835 | T | G | -0.2 | 0.041793 | 1.63E-06 | 3383 |
| class.Methanobacteria | rs894996 | A | C | 0.217 | 0.044908 | 1.88E-06 | 3582 |
| class.Methanobacteria | rs11018665 | T | A | 0.111 | 0.025052 | 6.52E-06 | 3698 |
| class.Methanobacteria | rs75208022 | T | C | -0.23 | 0.048763 | 5.92E-06 | 3203 |
| class.Methanobacteria | rs12825290 | G | C | -0.22 | 0.04935 | 6.08E-06 | 3144 |
| class.Methanobacteria | rs56131665 | A | G | 0.179 | 0.039298 | 6.18E-06 | 3572 |
| class.Methanobacteria | rs4257531 | A | G | 0.164 | 0.036496 | 7.44E-06 | 3162 |
| class.Methanobacteria | rs10424197 | A | G | -0.11 | 0.024752 | 9.28E-06 | 3698 |
| class.Methanobacteria | rs6508769 | C | T | 0.154 | 0.034452 | 8.23E-06 | 3319 |
| class.Methanobacteria | rs73068003 | T | G | -0.16 | 0.03518 | 8.45E-06 | 3692 |
| family.Methanobacteriaceae | rs12825290 | G | C | -0.22 | 0.04935 | 6.08E-06 | 3144 |
| family.Methanobacteriaceae | rs56131665 | A | G | 0.179 | 0.039298 | 6.18E-06 | 3572 |
| family.Methanobacteriaceae | rs11018665 | T | A | 0.111 | 0.025052 | 6.52E-06 | 3698 |
| family.Methanobacteriaceae | rs4257531 | A | G | 0.164 | 0.036496 | 7.44E-06 | 3162 |
| family.Methanobacteriaceae | rs10424197 | A | G | -0.11 | 0.024752 | 9.28E-06 | 3698 |
| family.Methanobacteriaceae | rs6508769 | C | T | 0.154 | 0.034452 | 8.23E-06 | 3319 |
| family.Methanobacteriaceae | rs73068003 | T | G | -0.16 | 0.03518 | 8.45E-06 | 3692 |
| family.Methanobacteriaceae | rs10202904 | G | T | -0.12 | 0.023536 | 3.01E-07 | 3695 |
| family.Methanobacteriaceae | rs73457410 | G | A | 0.215 | 0.04367 | 1.41E-06 | 3498 |
| family.Methanobacteriaceae | rs62241835 | T | G | -0.2 | 0.041793 | 1.63E-06 | 3383 |
| family.Methanobacteriaceae | rs894996 | A | C | 0.217 | 0.044908 | 1.88E-06 | 3582 |
| family.Methanobacteriaceae | rs75208022 | T | C | -0.23 | 0.048763 | 5.92E-06 | 3203 |
| genus.Adlercreutzia | rs7680684 | T | C | -0.08 | 0.01689 | 9.77E-07 | 8243 |
| genus.Adlercreutzia | rs2147798 | G | C | 0.092 | 0.019212 | 1.40E-06 | 8243 |
| genus.Adlercreutzia | rs80078995 | T | A | -0.11 | 0.023259 | 1.57E-06 | 8243 |
| genus.Adlercreutzia | rs2717140 | T | C | -0.12 | 0.025108 | 2.05E-06 | 8243 |
| genus.Adlercreutzia | rs9490822 | T | C | -0.07 | 0.015579 | 2.54E-06 | 8243 |
| genus.Adlercreutzia | rs6664405 | C | T | -0.1 | 0.021075 | 5.23E-06 | 8051 |
| genus.Adlercreutzia | rs12522517 | T | A | -0.1 | 0.023469 | 4.41E-06 | 8187 |
| genus.Adlercreutzia | rs13231526 | A | C | 0.143 | 0.031165 | 4.81E-06 | 8051 |
| genus.Adlercreutzia | rs1046175 | G | C | 0.113 | 0.025599 | 6.36E-06 | 8051 |
| genus.Adlercreutzia | rs55719207 | A | G | -0.07 | 0.015804 | 9.61E-06 | 8243 |
| genus.Adlercreutzia | rs9915817 | C | T | 0.075 | 0.016833 | 8.22E-06 | 8243 |
| genus.Adlercreutzia | rs11604400 | T | C | -0.1 | 0.023483 | 9.74E-06 | 8107 |
| genus.Collinsella | rs9541268 | A | C | 0.096 | 0.019731 | 8.79E-07 | 12914 |
| genus.Collinsella | rs2671662 | G | C | -0.06 | 0.011945 | 2.22E-06 | 14334 |
| genus.Collinsella | rs73052258 | A | G | 0.093 | 0.020266 | 1.72E-06 | 14334 |
| genus.Collinsella | rs2103510 | A | G | 0.079 | 0.016822 | 2.42E-06 | 14334 |
| genus.Collinsella | rs75672793 | G | A | -0.11 | 0.024052 | 6.14E-06 | 12601 |
| genus.Collinsella | rs10890671 | C | T | -0.05 | 0.011885 | 6.52E-06 | 14334 |
| genus.Collinsella | rs12921100 | T | A | 0.056 | 0.012702 | 8.23E-06 | 14334 |
| genus.Collinsella | rs62448871 | A | C | -0.05 | 0.012032 | 6.78E-06 | 14334 |
| genus.Collinsella | rs59414781 | G | C | 0.067 | 0.014977 | 9.15E-06 | 14334 |
| genus.Collinsella | rs1496626 | C | T | -0.07 | 0.01616 | 6.78E-06 | 14334 |
| genus.Collinsella | rs149807560 | A | C | -0.1 | 0.023584 | 7.10E-06 | 13473 |
| genus.Collinsella | rs11597285 | T | G | -0.05 | 0.012055 | 9.38E-06 | 14334 |
| genus.Phascolarctobacterium | rs6427992 | C | G | -0.07 | 0.013751 | 2.09E-06 | 11113 |
| genus.Phascolarctobacterium | rs75882962 | C | T | 0.097 | 0.019058 | 3.19E-07 | 10710 |
| genus.Phascolarctobacterium | rs56157888 | C | A | 0.095 | 0.019398 | 1.09E-06 | 10031 |
| genus.Phascolarctobacterium | rs56069061 | A | G | -0.11 | 0.023069 | 1.87E-06 | 11007 |
| genus.Phascolarctobacterium | rs74540770 | A | G | -0.12 | 0.025862 | 3.60E-06 | 9403 |
| genus.Phascolarctobacterium | rs76124218 | G | C | -0.16 | 0.034453 | 2.67E-06 | 7314 |
| genus.Phascolarctobacterium | rs12618201 | G | A | 0.064 | 0.01382 | 3.38E-06 | 11113 |
| genus.Phascolarctobacterium | rs74847270 | G | A | -0.1 | 0.0231 | 5.73E-06 | 11098 |
| genus.Phascolarctobacterium | rs28525131 | A | G | -0.12 | 0.026905 | 8.23E-06 | 10457 |
| genus.Phascolarctobacterium | rs1264476 | G | T | 0.077 | 0.016604 | 4.30E-06 | 11104 |
| genus.Phascolarctobacterium | rs7982713 | A | G | 0.073 | 0.01632 | 9.72E-06 | 10710 |
| genus.Phascolarctobacterium | rs11929846 | C | T | -0.07 | 0.015797 | 8.88E-06 | 11114 |
| genus.Prevotella9 | rs111509883 | C | T | 0.171 | 0.034762 | 1.24E-06 | 6960 |
| genus.Prevotella10 | rs2683313 | G | A | -0.07 | 0.01516 | 1.69E-06 | 10260 |
| genus.Prevotella11 | rs117271932 | G | A | 0.208 | 0.044039 | 2.82E-06 | 4878 |
| genus.Prevotella12 | rs10512344 | G | C | 0.247 | 0.054409 | 3.19E-06 | 3063 |
| genus.Prevotella13 | rs9428102 | G | A | -0.08 | 0.017607 | 4.62E-06 | 10264 |
| genus.Prevotella14 | rs746764 | C | T | -0.09 | 0.019324 | 2.04E-06 | 9385 |
| genus.Prevotella15 | rs16966465 | C | G | 0.074 | 0.016529 | 9.33E-06 | 10261 |
| genus.Prevotella16 | rs2104588 | C | T | 0.106 | 0.023773 | 8.13E-06 | 9268 |
| genus.Prevotella17 | rs11199734 | T | A | 0.077 | 0.016946 | 7.00E-06 | 10263 |
| genus.Prevotella18 | rs7232121 | C | G | 0.067 | 0.014423 | 3.76E-06 | 9790 |
| genus.Prevotella19 | rs11685699 | T | C | -0.14 | 0.029566 | 2.03E-06 | 8342 |
| genus.Prevotella20 | rs9613013 | A | G | 0.092 | 0.02027 | 6.10E-06 | 9790 |
| genus.Prevotella21 | rs2495052 | G | A | 0.084 | 0.018852 | 8.97E-06 | 9790 |
| genus.Prevotella22 | rs72815774 | C | T | -0.18 | 0.039307 | 8.78E-06 | 5728 |
| genus.Prevotella23 | rs4968431 | T | G | 0.064 | 0.014416 | 8.58E-06 | 10271 |
| genus.Prevotella24 | rs7976209 | C | T | -0.09 | 0.019795 | 7.28E-06 | 10263 |
| genus.Prevotella25 | rs12648235 | C | T | 0.079 | 0.017768 | 7.39E-06 | 10271 |
| genus.Prevotella26 | rs1304512 | A | G | 0.076 | 0.016588 | 5.29E-06 | 9790 |
| genus.Prevotella27 | rs7237249 | T | C | -0.08 | 0.018213 | 8.93E-06 | 10271 |
| genus.RuminococcaceaeUCG013 | rs4385846 | T | G | 0.06 | 0.013181 | 6.46E-06 | 16772 |
| genus.RuminococcaceaeUCG014 | rs12781711 | T | C | -0.07 | 0.011748 | 2.55E-08 | 16772 |
| genus.RuminococcaceaeUCG015 | rs12189346 | A | G | 0.068 | 0.014558 | 1.68E-06 | 16470 |
| genus.RuminococcaceaeUCG016 | rs75088940 | C | T | -0.09 | 0.020071 | 2.55E-06 | 15638 |
| genus.RuminococcaceaeUCG017 | rs16918863 | C | A | 0.111 | 0.024016 | 4.16E-06 | 12643 |
| genus.RuminococcaceaeUCG018 | rs76973485 | T | G | 0.195 | 0.041821 | 3.35E-06 | 5299 |
| genus.RuminococcaceaeUCG019 | rs12485353 | A | G | -0.06 | 0.013085 | 4.19E-06 | 16771 |
| genus.RuminococcaceaeUCG020 | rs9565219 | A | T | -0.05 | 0.01177 | 8.73E-06 | 16772 |
| genus.RuminococcaceaeUCG021 | rs1729063 | C | G | -0.05 | 0.012076 | 9.64E-06 | 16470 |
| genus.RuminococcaceaeUCG022 | rs7784330 | A | G | -0.05 | 0.011207 | 8.16E-06 | 16770 |
| genus.RuminococcaceaeUCG023 | rs11581881 | T | C | 0.066 | 0.014474 | 4.73E-06 | 15730 |
| genus.RuminococcaceaeUCG024 | rs2428106 | G | C | -0.05 | 0.01099 | 8.38E-06 | 16768 |
| genus.RuminococcaceaeUCG025 | rs2730183 | A | G | -0.05 | 0.010991 | 8.44E-06 | 16772 |
| genus.RuminococcaceaeUCG026 | rs9313055 | C | T | 0.105 | 0.023446 | 9.55E-06 | 15764 |
| genus.RuminococcaceaeUCG027 | rs12336782 | C | T | -0.09 | 0.018931 | 8.60E-06 | 16772 |
| genus.Slackia | rs8901 | T | C | 0.093 | 0.018681 | 6.07E-07 | 6063 |
| genus.Slackia | rs4492265 | G | A | -0.09 | 0.019166 | 2.41E-06 | 6063 |
| genus.Slackia | rs16894137 | T | C | -0.12 | 0.026305 | 2.71E-06 | 6063 |
| genus.Slackia | rs112764253 | A | T | 0.195 | 0.04116 | 3.40E-06 | 5634 |
| genus.Slackia | rs12440440 | G | A | 0.09 | 0.019058 | 2.63E-06 | 6063 |
| genus.Slackia | rs10409783 | G | A | 0.095 | 0.021124 | 7.70E-06 | 5941 |
| genus.Slackia | rs35156985 | C | T | -0.16 | 0.034808 | 8.06E-06 | 5965 |
| genus.Slackia | rs13339230 | G | C | 0.147 | 0.033064 | 7.42E-06 | 6063 |
| genus.Slackia | rs58767323 | C | G | -0.1 | 0.022708 | 4.60E-06 | 6063 |
| order.Methanobacteriales | rs10202904 | G | T | -0.12 | 0.023536 | 3.01E-07 | 3695 |
| order.Methanobacteriales | rs894996 | A | C | 0.217 | 0.044908 | 1.88E-06 | 3582 |
| order.Methanobacteriales | rs73457410 | G | A | 0.215 | 0.04367 | 1.41E-06 | 3498 |
| order.Methanobacteriales | rs62241835 | T | G | -0.2 | 0.041793 | 1.63E-06 | 3383 |
| order.Methanobacteriales | rs4257531 | A | G | 0.164 | 0.036496 | 7.44E-06 | 3162 |
| order.Methanobacteriales | rs75208022 | T | C | -0.23 | 0.048763 | 5.92E-06 | 3203 |
| order.Methanobacteriales | rs12825290 | G | C | -0.22 | 0.04935 | 6.08E-06 | 3144 |
| order.Methanobacteriales | rs56131665 | A | G | 0.179 | 0.039298 | 6.18E-06 | 3572 |
| order.Methanobacteriales | rs11018665 | T | A | 0.111 | 0.025052 | 6.52E-06 | 3698 |
| order.Methanobacteriales | rs6508769 | C | T | 0.154 | 0.034452 | 8.23E-06 | 3319 |
| order.Methanobacteriales | rs73068003 | T | G | -0.16 | 0.03518 | 8.45E-06 | 3692 |
| order.Methanobacteriales | rs10424197 | A | G | -0.11 | 0.024752 | 9.28E-06 | 3698 |
